# Genetic Structure and Gene Flows within Horses: A Genealogical Study at the French Population Scale

**DOI:** 10.1371/journal.pone.0061544

**Published:** 2013-04-22

**Authors:** Pauline Pirault, Sophy Danvy, Etienne Verrier, Grégoire Leroy

**Affiliations:** 1 AgroParisTech, Unité Mixte de Recherche 1313 Génétique Animale et Biologie Intégrative, Paris, France; 2 Institut Français du Cheval et de l'Equitation, Le Pin au Haras, France; 3 Institut National de la Recherche Agronomique, Unité Mixte de Recherche 1313 Génétique Animale et Biologie Intégrative, Domaine de Vilvert, Jouy-en-Josas, France; University of Sydney, United States of America

## Abstract

Since horse breeds constitute populations submitted to variable and multiple outcrossing events, we analyzed the genetic structure and gene flows considering horses raised in France. We used genealogical data, with a reference population of 547,620 horses born in France between 2002 and 2011, grouped according to 55 breed origins. On average, individuals had 6.3 equivalent generations known. Considering different population levels, fixation index decreased from an overall species *F_IT_* of 1.37%, to an average 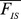 of −0.07% when considering the 55 origins, showing that most horse breeds constitute populations without genetic structure. We illustrate the complexity of gene flows existing among horse breeds, a few populations being closed to foreign influence, most, however, being submitted to various levels of introgression. In particular, Thoroughbred and Arab breeds are largely used as introgression sources, since those two populations explain together 26% of founder origins within the overall horse population. When compared with molecular data, breeds with a small level of coancestry also showed low genetic distance; the gene pool of the breeds was probably impacted by their reproducer exchanges.

## Introduction

The horse is a species raised for very diverse purposes. During the last 100 years, in the industrialized world, strong changes occurred in the ways of using horses: up to World War I, horses were mainly used for war, carriage and agricultural work; now, horses are mainly used for sport, leisure, hobby and even as a companion animal. These changes had two main consequences: first, the actual population size of racing and riding breeds have largely increased, whereas many draught breeds are now endangered; second, horse breeders have strongly modified their breeding goals. Another consequence is that outcrossing was commonly practiced for some breeds, and is still practiced, to improve performance of international and local populations. Distinction has to be made in relation to studbooks regulations. Indeed some studbooks are closed (e.g., French Trotter, Arab, and Thoroughbred breeds), others give allowance to introduce new gene lines and stallions from other breeds. As an example, the Anglo-Arab breed is a former cross between Arab and Thoroughbred breeds, used here because of their high performance in endurance and speed, respectively [1].

Several studies have been performed to assess the impact of outcrossing on a specific or a limited number of horse breeds, based on genealogical [2]–[5] or molecular approaches [2], [6]–[10]. Molecular tools are especially useful to measure genetic differentiation and distance between breeds, and assess a theoretical amount of admixture within a given population related to some geneflows [11]. However, they may still have a lack of precision, when considering the exact amount of gene flow at a given time scale in comparison to a documented pedigree data base. Yet completeness and correctness of genealogical information constitutes the main limitations of pedigree approaches [12]. Indeed, it is difficult to study past gene flows among a large number of breeds, since studbooks are generally independently established, from one breed to another, even if several indicators are available for that, such as probability of gene origins [13] or approximations of Wright-statistics [14].

This multiracial research was aimed at studying gene flows considering the whole French horse population, using the database of the French Horse Institute (Institut Français du Cheval et de l'Equitation, IFCE), which registers all the horses raised in France and some of their ancestors of foreign origin (between 2 and 3 generations on average). Among others, our goal was to explain how a breed can contribute to these flows or be affected by them. A comparison between breed genealogical and molecular distance indicators was also conducted.

## Materials and Methods

### Genealogical database

The entire French horse database SIRE (French Equine Information System), which includes, according to IFCE, between 90 and 95% of horses raised in France, was analyzed in this study. It includes 139 studbook designations, corresponding to breeds or breed subpopulations (varieties) defined according to national or international studbook rules. Those designations are categorized by the IFCE in three different breed groups: (1) Race and riding horses, (2) Pony breeds and (3) Draught horses.

To define a “reference population”, we chose the group of animals born in France from 2002 to 2011, which corresponded to a total of 732,176 animals, all breeds and designations considered. Based on equivalent complete generations (EqG) [4], we removed from this group animals without origins provided (183,366 horses), as well as 13 studbook designations with average EqG lesser than 2 generations (1,190 horses). Then, the genealogical database consisted in the reference population as defined above (547,620 horses born in France, for 97 studbook designations) plus all known ancestors of this population (360,862 horses, 71,547 of them being born outside France).

For simplicity sake, studbook designations with less than 200 individuals registered over the 2002–2011 period (corresponding to foreign breeds) were grouped together into three foreign “origins” according to their respective group: (i) Other foreign race or riding breeds (23 designations), (ii) Other foreign pony (3 designations) and (iii) Other foreign draught horse (1 designation). Studbook designations corresponding to the same breed were grouped together, with only two exceptions. The first exception was for the case of three Anglo-Arab designations, differentiated in their studbook rule according to the percentage of Arab genes within individuals. The second one was for the case of two subpopulations of the Welsh Pony breed, merging 2 and 4 Welsh designations according to their type (Pony or Cob/crossed individual). The French designation “AQPS” (“*Autre Que Pur-Sang*”, literally other than Thoroughbred”), which denotes racing horses related to Thoroughbred, but not recognized due to regulation reasons (Artificial Insemination, non-pure Thoroughbred…), was also considered as an independent origin. Finally the reference population corresponded to 55 breeds, varieties and groups of breeds defined here as “origins”.

### Probability of gene identity and gathering

We analyzed genetic structure first by computing average inbreeding *F_I_* and coancestry *C_IJ_* coefficients [15] for each subpopulation. Due to computing constraints for populations with a large actual population size, *C_IJ_* was estimated by averaging coancestry coefficients over 100,000 pairs of individuals randomly sampled within subpopulations *I* and *J*, respectively. In order to characterize genetic structure within a subpopulation *I*, we computed fixation index F_IS-I_ considering the following equation [16],
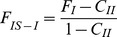
We also differentiated 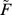 and 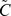 averaged within all subpopulations, and 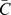 as coancestry averaged over the entire metapopulation [16], considering following equations, *N_I_* and *N_T_* being the population size of subpopulation *I* and the entire metapopulation, respectively,
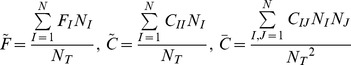
In order to compute *F*-statistics, we used the following equations,
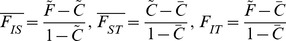

*F*-statistics indexes were calculated using the considering either the 3 horse breed groups (Race and riding horses, Pony and Draught horses), or the 55 breed origins as subpopulations.

Identity By Descent (IBD) coefficients such as *F* and *C* are considered to be very sensitive to incomplete pedigree information, (e.g., [12]). In order to study the relationships between breed origins, taking into account possible differences in pedigree knowledge, we used equivalent complete generations *EqG to* adjust coancestries between each couple of origins, according to the method developed by Cervantes et al.[17] to compute coancestry rates. Considering two origins *I* and *J*, two individuals *i* and *j* sampled within each one, *EqG_i_* and *EqG_j_* their respective equivalent complete generation and *C_ij_* their coancestry coancestry rate, *ΔC_ij_* can be computed using the following equation:
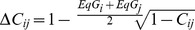
For each couple of origins, average coancestry rate 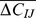 was computed by averaging coancestry rates over 100,000 individual pairs randomly sample within both origins.

A hierarchical clustering was carried out on the basis of the average of these coancestry rates computed among the 55 origins, using the Ward method, distances being determined on the basis of the coancestry rates (1−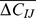), and the phenogram of relations being produced using the R *hclust* function.

Considering 32 horse breeds with genotype available from the Leroy *et al.* [6] study, we compared coancestry rates with Reynolds et al. [18] molecular distances computed for these 32 horse breeds.

### Probability of genes origin

On the basis of the hierarchical clustering results, origins were grouped to make a focus on gene flows existing among Race and riding horse populations. Pony and Draught horse origins were grouped into their respective breed group. The 15 Race and riding horse origins with reference population containing less than 5,000 horses and Certified race and riding origins were gathered into a single group (OTHERS), as well as the three American breeds (Quarter Horse, Paint Horse and Appaloosa). Finally, 11 Race and riding breeds and groups of breeds were studied, in relation with the other two horse groups (Pony and Draught horses).

Ancestral gene flows (parental and founder) were studied considering either the three horse groups (Race and riding horses, Pony and Draught horses) or the 13 groups, using probability of gene origins. The probability of gene origin is the probability for a gene taken at random within the reference population to come from an ancestor or founder [12]. We consider here a founder as an ancestor of the reference population without any known parent.

This study was performed using programs of PEDIG software ([19], http://www-sgqa.jouy.inra.fr/diffusions/htm) and our own FORTRAN routines (document S1).

## Results

### Demographical parameters and pedigree completeness

The 55 origins under study had different reference population sizes, ranging from 10 (Other foreign draught group breeds) to 109,551 individuals (French Trotter breed). If we consider the three breed groups, the Race and riding horse group had the largest reference population size with 339,574 horses. For the whole reference population, the pedigree knowledge was good, with an average *EqG* equal to 6.3 generations. Race and riding, Pony and Draught horse groups showed average *EqG* equal to 7.09, 5.2 and 4.93 respectively. According to Table 1, pedigree knowledge showed, however, a wide range of variation according to origins, *EqG* ranging from 2.01 (Other Foreign Draught Horse origin) to 8.83 (Thoroughbred).

**Table 1:** Demographic and genealogical indicators forthe 55 horse origins.

Breed group	Race and riding horses groups	Origin	Nr of horses (2002–2011)	EqG	F (%)	C (%)	F_IS_ (%)
**Race and riding**	**AA**	Anglo-Arab	6,760	7.69	1.92	1.72	0.2
		Complement Anglo-Arab	2,443	8.29	0.75	1.07	−0.33
		CrossedAnglo-Arab	2,587	7.21	0.67	1.06	−0.4
	**AQPS**	AQPS (Other Than Thoroughbred)	10,454	8.65	1.08	1.41	−0.33
	**AR**	Arab	14,293	7.3	3.38	1.69	1.73
	**CAM**	Camargue	5,871	2.87	3.19	1.07	2.14
	**FT**	French Trotter	109,551	7.14	2.24	2.61	*−0.38*
	**HBA**	Half Bred Arab	9,251	5.17	0.24	0.56	−0.31
	**MER**	Merens	5,035	6.05	5.32	5.64	−0.34
	**SF**	Selle Français	78,747	7.29	1.34	1.66	−0.33
	**THB**	Thoroughbred	50,919	8.83	1.81	1.94	−0.13
	**USA**	Paint Horse	2,386	4.7	0.27	0.36	−0.09
		Appaloosa	1,409	4.41	0.6	0.55	0.05
		Quarter Horse	2,543	4.82	0.87	0.6	0.28
	**OTHERS**	Arab-Barb	841	4.04	0.67	0.79	−0.12
		Barb	540	3.43	1.02	1.64	−0.63
		Certified race and riding origin	22,554	4.84	0.44	0.14	0.3
		Cream Horse	267	3.3	2.09	1.31	0.8
		Frisian	705	5.8	4.49	5.74	−1.32
		Henson	380	5.49	2.25	4.77	−2.65
		Icelandic Horse	942	5.24	1.88	1.41	0.47
		Lipizzan	465	5.65	2.03	3.57	−1.6
		Lusitano horse	3,558	5.8	3.43	2.41	1.04
		Other foreign race and riding breeds	627	5.47	1.04	0.45	0.6
		Royal Dutch Sport Horse	213	4.01	0.28	0.44	−0.16
		Shagya	453	5.92	1.79	3.14	−1.39
		Spanish Purebred	1,909	4.52	1.23	0.62	0.61
		Trakehner	237	6.08	1.28	2.19	−0.93
		Zangersheide	1,201	6.8	0.63	1.12	−0.5
**Pony**		Certified pony origin	14,404	4.31	0.31	0.18	0.13
		Connemara	5,433	6.23	3.68	3.84	−0.17
		Dartmoor	581	5.47	3.24	3.82	−0.61
		Fjord	2,191	5.24	0.96	1.59	−0.65
		French saddle pony	12,658	5.56	0.48	0.55	−0.07
		Haflinger	3,379	7.82	3.72	4.22	−0.52
		Highland	242	5.89	2.67	4.31	−1.72
		Landais	512	3.71	3.63	4.73	−1.16
		New-Forest	1,285	5.55	1.5	1.96	−0.47
		Other foreign pony	35	3.74	1.62	8.25	−7.23
		Pottok	3,030	2.22	0.91	0.44	0.47
		Shetland	5,105	5.52	1.52	0.64	0.89
		Welsh Cob	1,237	6.16	4.48	3.09	1.44
		Welsh Pony	1,081	5.62	2.71	1.64	1.09
**Draught horses**		Ardennais	7,512	3.56	0.68	0.6	0.09
		Auxois	1,209	3.7	0.75	1.21	−0.47
		Boulonnais	2,664	7.14	6.07	7.1	−1.11
		Breton	37,563	4.18	0.85	0.87	−0.02
		Certified draught horse origin	45,883	3.98	0.37	0.28	0.09
		Cob Normand	4,966	3.46	0.93	1.65	−0.73
		Comtois	44,394	7.19	2.48	2.37	0.12
		Franches-Montagnes	300	5.46	1.33	2.48	−1.18
		Other foreign draught horse	10	2.01	0.31	2.89	−2.65
		Percheron	12,870	3.75	0.69	0.87	−0.18
		Poitevin Mulassier	888	6.86	6.13	7.55	−1.53
		Trait du Nord	1,047	3.49	0.5	1.38	−0.89

EqG = Number of equivalent generations, F = inbreeding, C = coancestry, F_IS_ = fixation index.

### Inbreeding, coancestry and F-statistics

Within each breed group (Race and riding horses, Pony, Draught horses), average inbreeding was found to be equal to 1.79, 1.41 and 1.26% respectively, while coancestry was found to be equal to 0.49, 0.30 and 0.41% respectively. However, according to Table 2, average coancestry coefficients between horse groups were smaller than within-group coancestry coefficients (under 0.02%), underlining differentiation between horse groups.

**Table 2:** Inbreeding (F), coancestry (C) coefficients and fixation index F_IS_ for Race and riding, Pony and Draught horse breed groups.

Horse type	Number of horses (2002–2011)	F (%)	C (%)			F_IS_ (%)
			Race and riding	Pony	Draught	
Race and riding	340,171	1.79	0.49	0.02	0.00	1.30
Pony	48,143	1.41		0.30	0.00	1.11
Draught	159,306	1.26			0.41	0.85

0.00 is different from absolute zero.

By contrast, inbreeding and coancestry coefficients were not found to be so well differentiated when considering breed origins. *F* ranged from 0.24 (Half Bred Arab) to 6.13% (Poitevin Mulassier breed) and *C* ranged from 0.14 (Certified race and riding origin) to 8.25% (Other foreign pony). In general, higher values were found for breeds with small actual population size and higher *EqG*. The high coancestry level found for the Other foreign pony origin was, however, due to a sampling effect, with 20 of the 35 individuals sharing a common sire.

These contrasts between inbreeding and coancestry can be well illustrated when considering average F-statistics (Table 3). The higher value was found for overall species *F_IT_* (1.37%), a slightly lower value being found for subpopulation 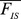 when considering breed groups (1.16%). This indicates a relative genetic structure remains within breed groups. By contrast, at the origin (i.e. breed) level, 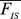 was found to be slightly negative (−0.07%), underlining that breeds constitute in general populations with almost no genetic structure. This was, however, not always the case, since a few origins showed *F_IS_* larger than 1% (Camargue and Arab breeds, the two Welsh origins and the Lusitano breed).

**Table 3:** Fixation indexes considering either the 3 breed groups or the 55 breed origins (%).

Category	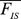 (%)	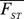 (%)	*F_IT_* (%)
3 breed groups	1.16	0.22	1.37
55 breedorigins	−0.07	1.44	

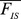
 = within variety fixation index, 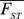 = between variety fixation index, FIT = global fixation index.

### Genetic relations and gene flows within and between horse populations

Over the 1485 average coefficients of coancestry computed across the 55 breed origins, 691 were different from zero, ranging from 10^−7^ to 1.44% (AQPS and Thoroughbred). Each origin showed at least 2 non zero coefficients of coancestry with other breed origins (*Table S3*).

**Figure 1:**
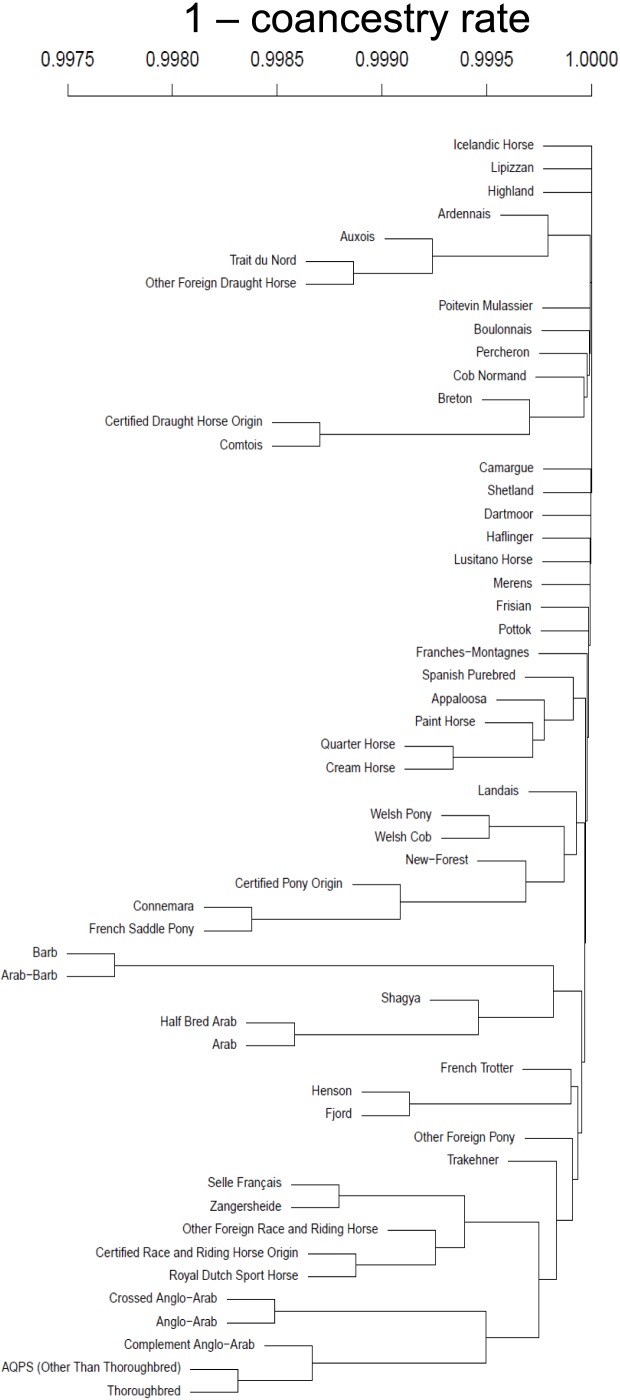
Current gene flows and founder origins for the three categories Race and riding horses (blue), Pony (green) and Draught Horses (pink). Number of individuals (2002–2011) in parenthesis.

The phenogram based on those coancestry rates allowed empirically assigning most of the origins into their respective breed group (Figure 1), some pony origins (Henson, Fjord), however, being found with Race and riding horse origins. Race and riding horse origins showed more contrasted relations than other breed groups, probably due to larger coancestry coefficients among origins, in relation to larger amounts of gene flow.

**Figure 2:**
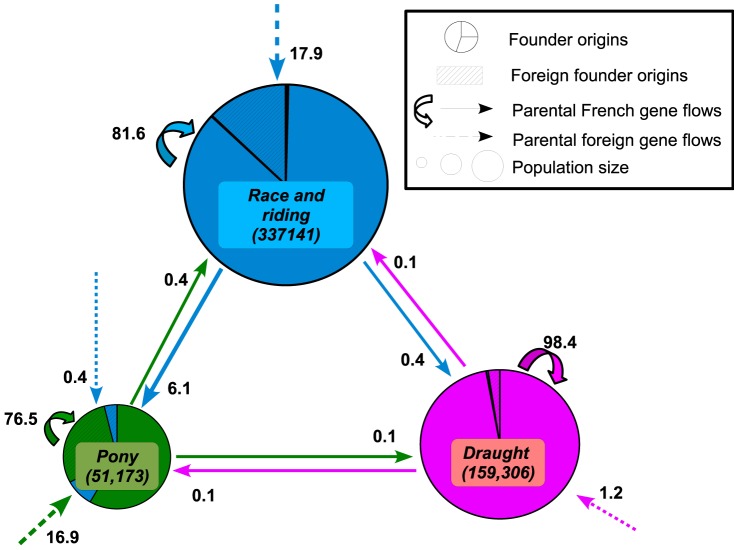
Phenogram of relations between the 55 breed origins, based on 1 – coancestry rate.

Figure 2 shows founder and parental gene flows between the 3 breed groups, origins and flows from individuals born in foreign countries being considered separately. Considering breed composition, Draught and Race and riding horses groups were found to be quite homogeneous with 99.5% of intern founder origins. By contrast, 12.4% of founder origins in the Pony breed group, were due to Race and riding horses. A more or less large foreign founder gene flow has to be noticed in each breed group (Race and riding horses: 12.9%, Pony: 29.3% and Draught horses: 2.4%) (see Table S1).

About current gene flows, 6.1% of the ponies had parents from the Race and riding horses group. A large number of parents seem to be born outside of France for Race and riding horses and Ponies, with animals of foreign country origin accounting for 17.9% and 16.9%, respectively.

Breeds were found to be submitted to various gene flows, the percentage of genetic variability being explained by external introgression ranging from almost 0 to 100% according to origins, considering either parents or founders (Table S2). In particular, 9 origins over the 55 showed no introgression, considering either parents or founders. The percentage of genetic variability being explained by parents and founders born in France ranged from 16 (Franche-Montagne, a breed of Swiss origin) to 100% and from almost 0.5 (Lusitano, a breed of Portugese origin) to 100%, respectively. The amount of parental introgression was larger when considering sire pathway (21.1% on average over breeds) than when considering dam pathway (14.7% on average over breeds). The Camargue breed was found to be the only origin with 100% of parents and founders born in France.

**Figure 3:**
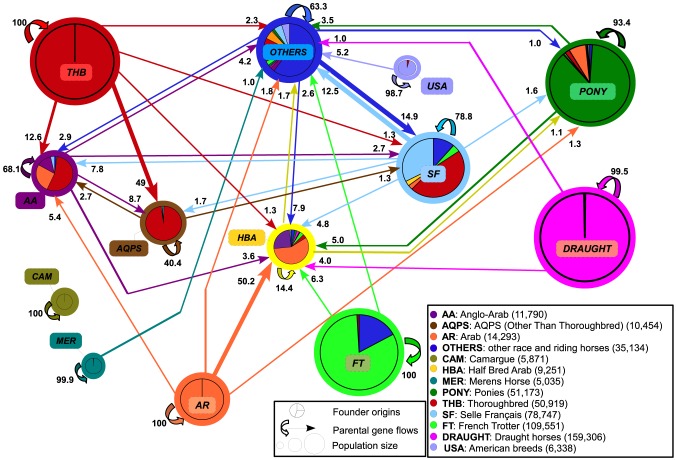
Current gene flows and founder origins for Race and riding horses populations. Parental gene flows under 1% are not presented here. Number of individuals (2002–2011) in parenthesis.

Figure 3 illustrates the complexity of gene flows among Race and riding horses. Only a small number of breeds seemed closed, namely Arab, Thoroughbred, Camargue and Merens breeds. On the contrary, composite horse populations were found to be more or less highly impacted by the former, i.e. founder, and current, i.e. parental, (AQPS, Half Blood Arab) external influences. Some breeds also showed an intermediate situation considering either the intensity or the time when cross-breeding had occurred, such as the French Trotter: 17% of the founders' origins were explained by Trotters from other countries, 100% of parental origins being, however, related to the French Trotter.

Breeds also seemed to be used with wide range of intensity as an introgression source. Thoroughbred and Arab breeds were in particular largely used for cross-breeding.While representing 4.2 and 15.1% of Race and riding horses population, these two breeds within Race and riding horses explained 5.6 and 18.3% of parental origins, and 7.9 and 32.9% of founder origins, respectively. At the species scale, both breeds explain altogether 26% of founder origins.

### Comparison between genealogical and molecular data

**Figure 4:**
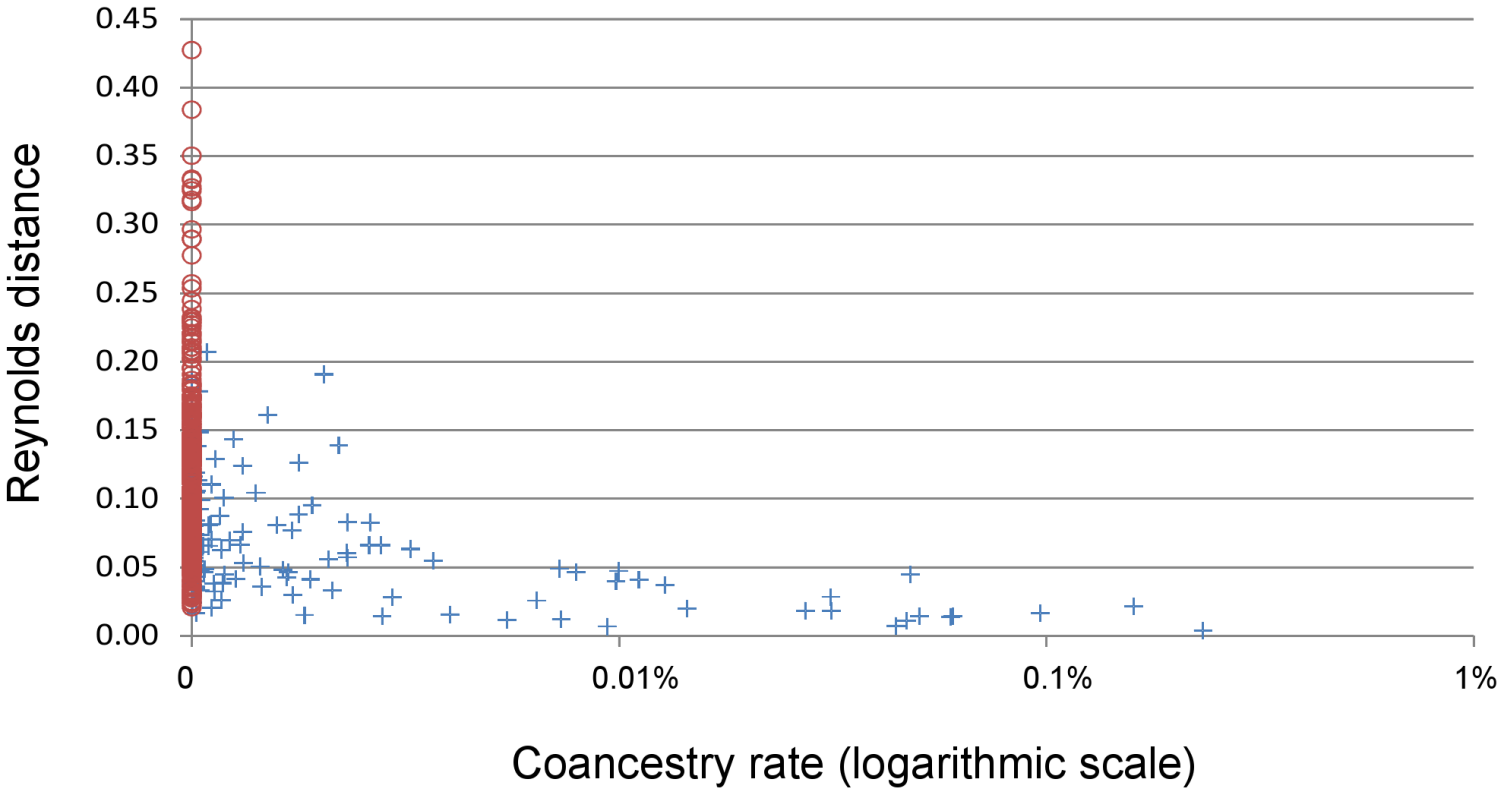
Relation between genealogical coancestry rates and molecular Reynold distances based on relation matrix of 32 horse breeds. Blue cross represent breed pairs with coancestry larger than 0, red circles represent breeds pairs with coancestry equal to 0.

Based on the comparison of 32 breeds in common with the study by Leroy et al. [6], coancestry rates and Reynolds' molecular distances were found to be negatively correlated (Spearman correlation of −0.39, P<0.0001 based on the Mantel test). One-hundred fifty-one coancestry rates over 496 were found different from 0, ranging from less than 10^−9^ to 0.2% per generation (Barb and Arab-Barb breeds). As illustrated by Figure 4, breeds with a minimum coancestry rate also showed low genetic distance, and for instance, each of the 22 pairs of breeds with coancestry rate larger than 0.005% showed genetic distance smaller than 0.05, i.e. less than half the average distance computed overall pairs (0.1).

## Discussion

### Genetic structure within the horse species

One of the aims of the study was to assess the different levels of genetic structure within the species, in a single country, on the basis of pedigree information. Computation of adapted F-statistics, from the species to the breed scale, constitutes an interesting tool for such a purpose. With a value of 1.37%, *F_IT_* computed for the species indicated a certain amount of genetic structure, as expected for a domestic species divided into several breeds. Considering the *F_IS_* estimated for the three breed groups, their respective values, lower than the overall *F_IT_*, but remaining larger than 0.8%, seem to confirm that they constitute a globally adequate classification, with, however, genetic structure remaining within each, as expected.

Finally the average 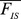 computed for the 55 origins (which roughly corresponds to the breed level), around −0.07%, also confirms that horse breeds constitute in general populations without genetic structure. Some exceptions were found, such as Arab, Camargue, Welsh and Lusitanian breeds, with *F_IS_* larger than 1%. For the Arab breed, this is probably related to inbreeding practices (intentional mating between close relatives) within the breed [2]. In the Spanish Arab Horse, Cervantes et al. [3] found indeed *F_IS_* close to 2%, in relation to preferential mating between relatives. By contrast, the existence of several subpopulations within the Welsh breed (Wahlund effect) explained the large *F_IS_* within the Welsh Cob and Welsh Pony origins (1.4 and 1.1% respectively). This structure level was, however, much smaller than if considering all Welsh studbook designations as one, *F_IS_* being then equal to 2.3% (data not shown). Similarly, the Wahlund effect was investigated in the Norikan draught horse by Druml et al. [20], in relation with coat color differentiation. For most of the other breeds, negative values of *F_IS_* were estimated, which could be explained by a limited population size (Highland pony and Henson breed for instance), a limited effective population size inducing a decrease of *F_IS_*, eventually to negative values [14]. Negative *F_IS_* is also expected for populations constituted by F1 crossed horses, with low inbreeding in comparison to coancestry (Half-Breed Arab).

When regarding studies using molecular markers to compute F-statistics on horse breeds with different origins [7], [8], [21], molecular *F_IT_* estimation was found much larger (around 12%), mainly in relation to higher *F_ST_* values, genealogical *F_ST_* being probably largely underestimated due to lack of pedigree information. In those three studies, breed *F_IS_* values were significantly (P = 0.0001) larger (on average 1.5% considering 73 breeds), in comparison to those determined in our study (on average −0.4% considering our 55 origins). By contrast, pedigree knowledge being important enough to assess genetic structure within most of the breeds studied in the present paper, such differences may be related to bias in molecular analysis, due to sampling or existence of null alleles when using microsatellites for instance, leading to an eventual overestimation of observed homozygosity. A more recent study using SNPs [10] showed mean *F_IS_* close to 0 (0.7% on average), higher *F_IS_* values being found in Arab, Shetland and Lusitano horse breeds, similarly to our study.

### Gene flow among horse breeds

This study allowed precisely measuring the current, or relatively recent, gene flows, existing among horse breeds. In agreement with *F_IS_* values, these gene flows exist preferentially within breed groups (Race and riding horses, Pony, draught horses), as investigated previously by Aberle et al. [5] in German Heavy horse breeds, or Cervantes et al. [3] within Spanish sport breeds. Yet as illustrated by Figure 2, some reproducers exchanges may occur, in particular from Race and riding horses to Pony breeds. Comparison between molecular and genealogical indicators confirms that breeds with regular reproducers exchanges also show molecular similarities (Figure 4). It is therefore not surprising that studies based on molecular markers, showed similarities between breeds related either to pony, draught horse, or race/riding horse breeds [7], [22], [23]. Those molecular studies provided however more complete view of breed genetic relationships, as they are not limited by pedigree knowledge.

Breeds themselves show contrasted patterns, considering either the way they impact or are impacted by introgression, the existence of exchange of reproducers between countries, as well as the evolution of gene flows over time (see for instance [24]). In relation to their studbook regulation, some of the origins considered here (e.g., Arab and Thoroughbred breeds) constitute populations closed to foreign influence, i.e. to introgression from other breeds or eventually also from individuals belonging to the same breeds but raised in another country (Camargue breed). Since no external genepool can be used to introduce some genetic variability within those populations, for breeds with limited population size, such as the Camargue breed, it appears important to limit erosion of their genetic diversity, through minimization of coancestry for instance [25]. However, according to our results, most of the horse breeds or populations seem submitted to more or less regular amounts of introgression. Those introgressions may have occurred during the former generations, with the founders belonging to external origins explaining on average 31% of genetic variability over the 55 origins, or on the contrary being continuous, 18% of parental origins being, on average over the 55 origins, related to individuals belonging to other breeds. Some horse origins are actually defined by the fact that they are constituted by first generation crossbred individuals, such as Half Bred Arab origin. It also has to be underlined that a limited part of horses raised in France do not belong to any specific breed. Certified origins or horses without known origins represent about 36% of horses registered in France between 2002 and 2011.This result was similar in Belgium for instance, where 36.5% of horses registered within the country are without origins (source: Belgian Horse Confederation, http://www.cbc-bcp.be, personal communication).

Results of the study show that outcrossing occurs mainly through the male pathway, in general using elite stallions which may be used in several breeds. As an example, Quidam de Revel, a stallion from the Selle Français breed, was used to produce in different riding horse breeds, specially German and Belgian ones (French Horse Riding Federation, http://www.ffecompet.ffe.com, 2013). Yet introgression is not exclusive of sire pathway, and in all the breeds submitted to introgression, outcrossing occurred both on sire and dam pathway.

Although pedigree analyses allow considering evolution of neutral diversity considering an entire population, they do have some limitations either due the extent of pedigree knowledge or to existence of pedigree errors (e.g., [26]). In our case, if on average individuals had 6.3 equivalent generation known, corresponding to 60 years (considering generation intervals equal to 9.6 years, as shown by Leroy et al. [27]), there is large differences between breeds and populations. For instance, while in most French breeds, systematic registration of foals within SIRE database (including their ancestors as far as possible) began in 1976, while for draught horses it took place in 1988, explaining lower pedigree knowledge for those breeds. As a consequence, time scale considered for IBD coefficients and probability of gene origins is not the same across populations (note it is however corrected for coancestry rates). This may lead to some bias [5] and the influence of foreign breeds (Arab and Thoroughbred breeds for instance) were probably underestimated, especially for breeds with poor pedigree knowledge [3], as founders considered in the study were probably already impacted by gene flows from those two breeds, due to former outcrossing events. The existence of pedigree errors, especially if linked with unofficial outcrossing events, may also have led to some bias in our results. It has however to be stated that paternity testing, which allow to limit such inaccuracies, is widely used in horse since more than twenty five years. In France, parentage control has been developed since the mid-seventies (first through blood typing), and is made systematically in some breeds since 1988 [2]. Since 2001, about 33 000 horses (i.e. more than 50% of the number of foals with origin identified) are genotyped for parentage control each year in France, reducing drastically the extent of pedigree errors. To conclude, it has to be stated that, when pedigree knowledge is not limiting, genealogical analyze may have some advantage in comparison to molecular data as (i) entire populations are considered, (ii) it may give accurate results for a defined generation (such as parental one here) and time scale.

The results of this study may lead to several ascertainments and recommendations regarding horse breeds management. Among others, it clearly underlines a current and large use of the Thoroughbred gene pool (and the Arab gene pool to a lesser extent) at least at the French population scale. As an example, from a genetic point of view, AQPS constitutes a population that is difficult to distinguish from Thoroughbreds, the latter breed explaining 97.3% of its founder origins (Table S4). To a lesser extent, the Thoroughbred constitutes also by far the main origin for the Selle Français and Anglo-Arab breeds (46.7 and 54.4% of founder origins, respectively). Such a result is in agreement with a previous study showing the large use of Thoroughbreds in outcrossing [28]. These two breeds can therefore be considered already as half Thoroughbred from a genetic point of view, previous studies based on molecular results suggesting that those breeds could be genetically even closer [7]. If breeders of these populations want to consider these populations as distinguished from the Thoroughbred, one can recommend to better monitor outcrossing in the future. Note, however, that our approach was based on evolution of neutral variability. In relation to the wide range of selection goals, populations could be more differentiated when considering selected genome areas. Our results also allow us to underline that the different subpopulations of the Welsh breed can be classified into two categories in relation to their type.

## Conclusion

Outcrossing constitutes a common practice within domestic animals, and has been punctually investigated for a given breed [28], [29]. This is the first study that makes a pedigree analysis considering individuals raised in a country whatever the breed. It is therefore possible to illustrate the complexity and the diversity of gene flows existing within a given domestic species. The genealogical approach is not as accurate as molecular analysis to measure genetic similarity between breeds, yet when enough pedigree knowledge is available, it provides accurate results about breed structure and recent gene flows, providing, among others, useful information for gene association studies [30]. These approaches could be improved by gathering pedigree data from foreign studbooks, underlining eventual differences in breeding practice according to countries, and allowing adequate recommendations to be given regarding management of genetic diversity for international breeds.

## Supporting Information

Table S1 **French and foreign founder origins of the three breed types: Race and riding horses, Pony and Draught horses.**
(DOCX)

Table S2 **Parental and founder origins for the 55 breed origins (%).**
(DOCX)

Table S3 **coancestry coefficients among the 55 breed origins (%).** (0.00 correspond to values different from absolute zero).
(DOCX)

Table S4 **Founder origins of the race and riding breeds and groups of breeds (%).**
(DOCX)

Document S1 **Program source code.**
(DOCX)
