# Supplementary material for: Genetic Structure and Gene Flows within Horses: A Genealogical Study at the French Population Scale
Source: PLoS One. 2013 Apr 22;8(4):e61544. doi: 10.1371/journal.pone.0061544 (PMC3632587; doi:10.1371/journal.pone.0061544)
Supplement: Table S1 — French and foreign founder origins of the three breed types: Race and riding horses, Pony and Draught horses. (DOCX) [file pone.0061544.s001.docx]

| **Breed type** | **Race and riding FR (%)** | **Pony FR (%)** | **Draught FR (%)** | **Race and riding  FO (%)** | **Pony FO (%)** | **Draught FO (%)** |
| --- | --- | --- | --- | --- | --- | --- |
| **Race and riding horses** | 86.58 | 0.24 | 0.16 | 12.90 | 0.11 | 0.01 |
| **Pony** | 8.58 | 58.20 | 0.11 | 3.81 | 29.29 | 0.00 |
| **Draught horses** | 0.37 | 0.04 | 97.19 | 0.02 | 0.01 | 2.37 |

**Table S1. French and foreign founder origins of the three breed types: Race and riding horses, Pony and Draught horses.**

FR : Horses born in France, FO : Horses born in foreign countries
