# Supplementary material for: Genetic Structure and Gene Flows within Horses: A Genealogical Study at the French Population Scale
Source: PLoS One. 2013 Apr 22;8(4):e61544. doi: 10.1371/journal.pone.0061544 (PMC3632587; doi:10.1371/journal.pone.0061544)
Supplement: Table S2 — Parental and founder origins for the 55 breed origins (%). (DOCX) [file pone.0061544.s002.docx]

**Table S2 Parental and founder origins for the 55 breed origins (%).**

| **Breed group** | **Race and riding horses groups** | **Origin** | **Parental intra origins** | | | **French parents** | **Founder intra origins** | **French founders** |
| --- | --- | --- | --- | --- | --- | --- | --- | --- |
|  |  |  | **Total** | **Sire** | **Dam** |  |  |  |
| **Race and riding** | **AA** | Anglo-Arab | 79.4 | 73.6 | 85.2 | 97.2 | 15.6 | 90.8 |
|  |  | Complement Anglo-Arab | 31.0 | 6.7 | 55.4 | 82.3 | 0 | 89.6 |
|  |  | CrossedAnglo-Arab | 0.4 | 0.2 | 0.6 | 89.7 | 0 | 90.0 |
|  | **AQPS** | AQPS (Other Than Thoroughbred) | 40.4 | 2.6 | 78.2 | 76.9 | 0 | 88.5 |
|  | **AR** | Arab | 100 | 100 | 100 | 79.1 | 100 | 42.1 |
|  | **CAM** | Camargue | 100 | 100 | 100 | 100 | 100 | 100 |
|  | **FT** | French Trotter | 100 | 100 | 100 | 98.3 | 82.1 | 97.8 |
|  | **HBA** | Half Bred Arab | 14.4 | 0.4 | 28.4 | 92.5 | 24.8 | 64.6 |
|  | **MER** | Merens | 99.9 | 100.0 | 99.7 | 98.5 | 99.3 | 100 |
|  | **SF** | Selle Français | 78.8 | 70.5 | 87.0 | 85.3 | 32.6 | 92.9 |
|  | **THB** | Thoroughbred | 100 | 100 | 100 | 52 | 100 | 83.6 |
|  | **USA** | Paint Horse | 78.5 | 87.6 | 69.4 | 48.5 | 30.9 | 53.2 |
|  |  | Appaloosa | 89.1 | 87.9 | 90.3 | 61.2 | 62.7 | 40.1 |
|  |  | Quarter Horse | 99.5 | 99.9 | 99.2 | 37.8 | 96.5 | 17.8 |
|  | **OTHERS** | Arab-Barb | 60.8 | 38.5 | 83.1 | 77.4 | 26.1 | 45.1 |
|  |  | Barb | 100 | 100 | 100 | 71.4 | 100 | 42.2 |
|  |  | Certified race and riding origin | 8.1 | 1.9 | 14.4 | 70.4 | 23.6 | 82.8 |
|  |  | Cream Horse | 84.8 | 86.8 | 82.9 | 93.4 | 67.3 | 83.9 |
|  |  | Frisian | 100 | 100 | 100 | 26.7 | 99.8 | 99.3 |
|  |  | Henson | 97.4 | 95.0 | 99.7 | 100 | 25.4 | 96.0 |
|  |  | Icelandic Horse | 100 | 100 | 100 | 62.3 | 100 | 48.9 |
|  |  | Lipizzan | 100 | 100 | 100 | 62.3 | 97.5 | 61.7 |
|  |  | Lusitano horse | 100 | 100 | 100 | 51.2 | 100 | 0.5 |
|  |  | Other foreign race and riding breeds | 75.5 | 77.1 | 73.9 | 33.6 | 51.5 | 89.2 |
|  |  | Royal Dutch Sport Horse | 68.5 | 65.5 | 71.4 | 67.4 | 30.7 | 91.9 |
|  |  | Shagya | 97.1 | 95.4 | 98.9 | 43.9 | 68.8 | 53.8 |
|  |  | Spanish Purebred | 100 | 100 | 100 | 45.7 | 98.6 | 95.1 |
|  |  | Trakehner | 95.4 | 97.9 | 92.8 | 20.3 | 70.4 | 23.0 |
|  |  | Zangersheide | 9.1 | 15.7 | 2.6 | 36.2 | 0.3 | 88.6 |
| **Pony** |  | Certified pony origin | 6.4 | 0.7 | 12.1 | 88.5 | 27.8 | 71.9 |
|  |  | Connemara | 100 | 100 | 100 | 88.6 | 97.9 | 31.1 |
|  |  | Dartmoor | 99.9 | 100 | 99.8 | 76.4 | 100 | 52.4 |
|  |  | Fjord | 100 | 100 | 100 | 78.4 | 100 | 88.1 |
|  |  | French saddle pony | 48.0 | 36.0 | 60.0 | 87.3 | 8.1 | 56.3 |
|  |  | Haflinger | 100 | 100 | 100 | 62.3 | 100 | 83.6 |
|  |  | Highland | 100 | 100 | 100 | 81 | 100 | 52.1 |
|  |  | Landais | 99.3 | 99.6 | 99.0 | 99.9 | 88.4 | 93.6 |
|  |  | New-Forest | 98.2 | 97.2 | 99.3 | 66.3 | 100 | 45.8 |
|  |  | Other foreign pony | 93.8 | 100.0 | 87.5 | 49.8 | 92.2 | 76.5 |
|  |  | Pottok | 97.5 | 95.0 | 100 | 99.3 | 96 | 97.9 |
|  |  | Shetland | 100 | 100 | 100 | 70.7 | 99.9 | 99.9 |
|  |  | Welsh Cob | 84.2 | 84.4 | 84.1 | 64.6 | 72.7 | 29.1 |
|  |  | Welsh Pony | 99.8 | 99.8 | 99.8 | 56.4 | 96.4 | 32.6 |
| **Draught horses** |  | Ardennais | 97.8 | 96.6 | 99.1 | 94.3 | 93 | 91.4 |
|  |  | Auxois | 77.3 | 59.1 | 95.4 | 90.5 | 9.8 | 87.3 |
|  |  | Boulonnais | 99.6 | 99.3 | 99.9 | 99.8 | 99 | 99.8 |
|  |  | Breton | 99.1 | 99.6 | 98.6 | 99.9 | 95 | 100 |
|  |  | Certified draught horse origin | 40.2 | 0.6 | 79.9 | 99.4 | 28.3 | 98.4 |
|  |  | Cob Normand | 94.2 | 100 | 88.3 | 100 | 80.9 | 100 |
|  |  | Comtois | 91.6 | 100 | 83.1 | 100 | 86.5 | 97 |
|  |  | Franches-Montagnes | 99.8 | 100 | 99.7 | 16.1 | 91.3 | 90.5 |
|  |  | Other foreign draught horse | 95.0 | 90.0 | 100 | 100 | 73.8 | 89.4 |
|  |  | Percheron | 99.5 | 100 | 99.0 | 97.8 | 97.6 | 95.2 |
|  |  | Poitevin Mulassier | 99.7 | 100 | 99.3 | 99.8 | 98.3 | 99.9 |
|  |  | Trait du Nord | 87.1 | 77.6 | 96.7 | 70.7 | 71.2 | 70.6 |
