# Supplementary material for: Genetic Structure and Gene Flows within Horses: A Genealogical Study at the French Population Scale
Source: PLoS One. 2013 Apr 22;8(4):e61544. doi: 10.1371/journal.pone.0061544 (PMC3632587; doi:10.1371/journal.pone.0061544)
Supplement: Table S3 — coancestry coefficients among the 55 breed origins (%). (0.00 correspond to values different from absolute zero). (DOCX) [file pone.0061544.s003.docx]

**Table S3: coancestry coefficients among the 55 breed origins (%)** (0.00 correspond to values different from absolute zero).

| **code** | **1** | **2** | **3** | **4** | **5** | **6** | **7** | **8** | **9** | **10** | **11** | **12** | **13** | **14** | **15** | **16** | **17** | **18** | **19** | **20** | **21** | **22** | **23** | **24** | **25** | **26** | **27** | **28** |
| --- | --- | --- | --- | --- | --- | --- | --- | --- | --- | --- | --- | --- | --- | --- | --- | --- | --- | --- | --- | --- | --- | --- | --- | --- | --- | --- | --- | --- |
| **1** | 1.72 | 0.74 | 1.13 | 0.37 | 0.46 | 0 | 0.00 | 0.25 | 0.00 | 0.24 | 0.34 | 0.00 | 0.01 | 0.00 | 0.08 | 0 | 0.19 | 0.01 | 0 | 0.11 | 0 | 0 | 0 | 0.06 | 0.06 | 0.05 | 0 | 0.19 |
| **2** |  | 1.07 | 0.47 | 1.09 | 0.09 | 0 | 0.00 | 0.07 | 0.00 | 0.26 | 1.16 | 0.01 | 0.01 | 0.01 | 0.02 | 0 | 0.15 | 0.01 | 0 | 0.11 | 0 | 0 | 0 | 0.07 | 0.09 | 0.00 | 0 | 0.14 |
| **3** |  |  | 1.06 | 0.24 | 0.26 | 0 | 0.03 | 0.18 | 0.00 | 0.55 | 0.21 | 0.00 | 0.01 | 0.00 | 0.06 | 0 | 0.24 | 0.01 | 0 | 0.11 | 0 | 0 | 0 | 0.10 | 0.13 | 0.05 | 0 | 0.17 |
| **4** |  |  |  | 1.41 | 0.01 | 0 | 0.00 | 0.03 | 0.00 | 0.26 | 1.45 | 0.01 | 0.02 | 0.01 | 0.00 | 0 | 0.13 | 0.00 | 0 | 0.10 | 0 | 0 | 0 | 0.07 | 0.09 | 0.00 | 0 | 0.11 |
| **5** |  |  |  |  | 1.69 | 0 | 0 | 0.86 | 0 | 0.02 | 0 | 0 | 0.02 | 0 | 0.27 | 0 | 0.08 | 0.08 | 0 | 0.02 | 0 | 0 | 0 | 0.02 | 0.00 | 0.39 | 0 | 0.02 |
| **6** |  |  |  |  |  | 1.07 | 0 | 0.00 | 0 | 0.00 | 0 | 0 | 0 | 0 | 0 | 0 | 0.01 | 0 | 0 | 0 | 0 | 0 | 0 | 0 | 0 | 0 | 0 | 0 |
| **7** |  |  |  |  |  |  | 2.61 | 0.09 | 0.01 | 0.05 | 0.00 | 0.00 | 0.00 | 0.00 | 0 | 0 | 0.08 | 0.00 | 0 | 0.12 | 0 | 0 | 0 | 0.11 | 0.00 | 0 | 0 | 0.00 |
| **8** |  |  |  |  |  |  |  | 0.56 | 0.03 | 0.04 | 0.03 | 0.00 | 0.02 | 0.00 | 0.18 | 0.00 | 0.06 | 0.06 | 0.00 | 0.02 | 0.00 | 0.00 | 0.00 | 0.02 | 0.01 | 0.26 | 0.00 | 0.02 |
| **9** |  |  |  |  |  |  |  |  | 5.64 | 0.00 | 0 | 0 | 0 | 0 | 0 | 0 | 0.09 | 0.00 | 0 | 0.00 | 0 | 0 | 0 | 0.01 | 0.00 | 0 | 0 | 0 |
| **10** |  |  |  |  |  |  |  |  |  | 1.66 | 0.25 | 0.00 | 0.00 | 0.00 | 0.00 | 0 | 0.39 | 0.01 | 0 | 0.15 | 0 | 0 | 0.00 | 0.24 | 0.35 | 0.00 | 0.00 | 0.06 |
| **11** |  |  |  |  |  |  |  |  |  |  | 1.94 | 0.02 | 0.02 | 0.01 | 0 | 0 | 0.13 | 0.00 | 0 | 0.10 | 0 | 0 | 0 | 0.08 | 0.10 | 0 | 0 | 0.12 |
| **12** |  |  |  |  |  |  |  |  |  |  |  | 0.36 | 0.03 | 0.16 | 0 | 0 | 0.01 | 0.01 | 0 | 0.00 | 0 | 0 | 0 | 0.00 | 0.00 | 0 | 0 | 0.00 |
| **13** |  |  |  |  |  |  |  |  |  |  |  |  | 0.55 | 0.08 | 0.00 | 0 | 0.02 | 0.01 | 0 | 0.00 | 0 | 0 | 0 | 0.00 | 0.00 | 0.01 | 0 | 0.00 |
| **14** |  |  |  |  |  |  |  |  |  |  |  |  |  | 0.60 | 0 | 0 | 0.02 | 0.02 | 0 | 0.00 | 0 | 0 | 0 | 0.00 | 0.00 | 0 | 0 | 0.00 |
| **15** |  |  |  |  |  |  |  |  |  |  |  |  |  |  | 0.79 | 0.83 | 0.03 | 0.02 | 0 | 0.00 | 0 | 0 | 0 | 0.00 | 0.00 | 0.08 | 0 | 0.00 |
| **16** |  |  |  |  |  |  |  |  |  |  |  |  |  |  |  | 1.64 | 0.02 | 0 | 0 | 0 | 0 | 0 | 0 | 0 | 0 | 0 | 0 | 0 |
| **17** |  |  |  |  |  |  |  |  |  |  |  |  |  |  |  |  | 0.14 | 0.02 | 0.04 | 0.06 | 0.00 | 0.01 | 0.06 | 0.09 | 0.12 | 0.03 | 0.01 | 0.04 |
| **18** |  |  |  |  |  |  |  |  |  |  |  |  |  |  |  |  |  | 1.31 | 0 | 0.00 | 0 | 0 | 0.02 | 0.00 | 0.00 | 0.02 | 0.02 | 0.00 |
| **19** |  |  |  |  |  |  |  |  |  |  |  |  |  |  |  |  |  |  | 5.74 | 0 | 0 | 0 | 0 | 0.19 | 0 | 0 | 0 | 0 |
| **20** |  |  |  |  |  |  |  |  |  |  |  |  |  |  |  |  |  |  |  | 4.77 | 0 | 0 | 0 | 0.03 | 0.03 | 0.00 | 0 | 0.02 |
| **21** |  |  |  |  |  |  |  |  |  |  |  |  |  |  |  |  |  |  |  |  | 1.41 | 0 | 0 | 0 | 0 | 0 | 0 | 0 |
| **22** |  |  |  |  |  |  |  |  |  |  |  |  |  |  |  |  |  |  |  |  |  | 3.57 | 0 | 0 | 0 | 0 | 0 | 0 |
| **23** |  |  |  |  |  |  |  |  |  |  |  |  |  |  |  |  |  |  |  |  |  |  | 2.41 | 0.04 | 0 | 0 | 0.00 | 0 |
| **24** |  |  |  |  |  |  |  |  |  |  |  |  |  |  |  |  |  |  |  |  |  |  |  | 0.45 | 0.14 | 0.01 | 0 | 0.06 |
| **25** |  |  |  |  |  |  |  |  |  |  |  |  |  |  |  |  |  |  |  |  |  |  |  |  | 0.44 | 0.00 | 0 | 0.12 |
| **26** |  |  |  |  |  |  |  |  |  |  |  |  |  |  |  |  |  |  |  |  |  |  |  |  |  | 3.14 | 0 | 0.00 |
| **27** |  |  |  |  |  |  |  |  |  |  |  |  |  |  |  |  |  |  |  |  |  |  |  |  |  |  | 0.62 | 0 |
| **28** |  |  |  |  |  |  |  |  |  |  |  |  |  |  |  |  |  |  |  |  |  |  |  |  |  |  |  | 2.19 |

1: Anglo-Arab; 2: Complement Anglo-Arab; 3: Crossed Anglo-Arab; 4: AQPS (Other Than Thoroughbred) 5: Arab; 6: Camargue; 7: French Trotter; 8: Half Bred Arab; 9: Merens; 10: Selle Français; 11: Thoroughbred; 12: Paint Horse; 13: Appaloosa; 14: Quarter Horse; 15: Arab-Barb 16: Barb 17: Certified race and riding horse; 18: Cream Horse;

| **code** | **29** | **30** | **31** | **32** | **33** | **34** | **35** | **36** | **37** | **38** | **39** | **40** | **41** | **42** | **43** | **44** | **45** | **46** | **47** | **48** | **49** | **50** | **51** | **52** | **53** | **54** | **55** |
| --- | --- | --- | --- | --- | --- | --- | --- | --- | --- | --- | --- | --- | --- | --- | --- | --- | --- | --- | --- | --- | --- | --- | --- | --- | --- | --- | --- |
| **1** | 0.17 | 0.07 | 0.00 | 0 | 0 | 0.09 | 0.00 | 0 | 0.01 | 0 | 0.01 | 0.01 | 0 | 0.01 | 0.00 | 0 | 0 | 0.00 | 0.00 | 0.00 | 0.00 | 0.00 | 0.00 | 0 | 0 | 0 | 0 |
| **2** | 0.18 | 0.04 | 0.00 | 0 | 0 | 0.03 | 0.00 | 0 | 0.00 | 0 | 0.01 | 0.00 | 0 | 0.01 | 0.00 | 0 | 0 | 0.00 | 0.00 | 0.00 | 0.00 | 0.00 | 0.00 | 0 | 0 | 0 | 0 |
| **3** | 0.33 | 0.07 | 0.00 | 0 | 0 | 0.07 | 0.00 | 0 | 0.01 | 0.00 | 0.01 | 0.01 | 0 | 0.01 | 0.00 | 0 | 0 | 0.00 | 0.00 | 0.00 | 0.00 | 0.00 | 0.01 | 0 | 0 | 0 | 0 |
| **4** | 0.19 | 0.03 | 0.00 | 0 | 0 | 0.02 | 0 | 0 | 0.00 | 0 | 0.01 | 0.00 | 0 | 0.01 | 0.00 | 0 | 0 | 0.00 | 0.00 | 0.00 | 0.00 | 0.00 | 0.00 | 0 | 0 | 0 | 0 |
| **5** | 0.01 | 0.08 | 0.01 | 0 | 0 | 0.20 | 0.00 | 0 | 0.05 | 0 | 0.00 | 0.03 | 0 | 0.00 | 0.00 | 0 | 0 | 0.01 | 0.00 | 0.00 | 0.00 | 0.00 | 0.00 | 0 | 0 | 0 | 0 |
| **6** | 0 | 0.00 | 0 | 0 | 0 | 0.00 | 0 | 0 | 0 | 0 | 0 | 0 | 0 | 0.00 | 0 | 0 | 0 | 0 | 0 | 0 | 0 | 0 | 0 | 0 | 0 | 0 | 0 |
| **7** | 0.03 | 0.04 | 0 | 0 | 0 | 0.01 | 0 | 0 | 0.00 | 0 | 0.00 | 0.00 | 0 | 0.00 | 0 | 0 | 0 | 0 | 0.00 | 0.01 | 0.00 | 0.00 | 0.00 | 0 | 0 | 0 | 0 |
| **8** | 0.03 | 0.06 | 0.02 | 0.00 | 0.00 | 0.13 | 0.01 | 0.00 | 0.03 | 0.00 | 0.00 | 0.02 | 0 | 0.01 | 0.00 | 0.00 | 0.00 | 0.06 | 0.00 | 0.00 | 0.00 | 0.01 | 0.00 | 0.00 | 0.00 | 0.00 | 0.00 |
| **9** | 0.00 | 0.01 | 0 | 0 | 0 | 0.00 | 0 | 0 | 0 | 0 | 0 | 0 | 0 | 0.00 | 0 | 0 | 0 | 0 | 0.00 | 0.02 | 0.00 | 0 | 0 | 0 | 0 | 0 | 0 |
| **10** | 0.85 | 0.07 | 0.00 | 0 | 0.00 | 0.05 | 0 | 0 | 0.00 | 0.00 | 0.01 | 0.00 | 0 | 0.03 | 0.00 | 0 | 0 | 0.00 | 0.00 | 0.00 | 0.00 | 0.00 | 0.04 | 0 | 0 | 0 | 0 |
| **11** | 0.19 | 0.03 | 0 | 0 | 0 | 0.01 | 0 | 0 | 0.00 | 0 | 0.01 | 0.00 | 0 | 0.01 | 0.00 | 0 | 0 | 0 | 0.00 | 0.00 | 0.00 | 0.00 | 0.00 | 0 | 0 | 0 | 0 |
| **12** | 0.00 | 0.00 | 0 | 0 | 0 | 0.00 | 0 | 0 | 0.00 | 0 | 0.00 | 0.00 | 0 | 0.00 | 0.00 | 0 | 0 | 0 | 0.00 | 0.00 | 0.00 | 0 | 0.00 | 0 | 0 | 0 | 0 |
| **13** | 0.00 | 0.00 | 0.00 | 0 | 0 | 0.00 | 0.00 | 0 | 0.00 | 0 | 0.00 | 0.00 | 0 | 0.00 | 0.00 | 0 | 0 | 0.00 | 0.00 | 0.00 | 0.00 | 0.00 | 0.00 | 0 | 0 | 0 | 0 |
| **14** | 0.00 | 0.00 | 0 | 0 | 0 | 0.00 | 0 | 0 | 0.00 | 0 | 0.00 | 0.00 | 0 | 0.00 | 0.00 | 0 | 0 | 0 | 0.00 | 0.00 | 0.00 | 0.00 | 0.00 | 0 | 0 | 0 | 0 |
| **15** | 0.00 | 0.02 | 0.00 | 0 | 0 | 0.04 | 0.00 | 0 | 0.02 | 0 | 0.00 | 0.01 | 0 | 0.00 | 0.00 | 0 | 0 | 0.00 | 0.00 | 0.00 | 0.00 | 0.00 | 0.00 | 0 | 0 | 0 | 0 |
| **16** | 0 | 0.00 | 0 | 0 | 0 | 0.00 | 0 | 0 | 0 | 0 | 0 | 0 | 0 | 0 | 0 | 0 | 0 | 0 | 0 | 0.00 | 0 | 0 | 0 | 0 | 0 | 0 | 0 |
| **17** | 0.28 | 0.03 | 0.03 | 0.00 | 0.01 | 0.04 | 0.01 | 0.00 | 0.01 | 0.01 | 0.01 | 0.00 | 0.00 | 0.02 | 0.01 | 0.00 | 0.00 | 0.01 | 0.00 | 0.00 | 0.00 | 0.00 | 0.01 | 0.00 | 0.00 | 0.00 | 0.00 |
| **18** | 0.00 | 0.01 | 0.00 | 0 | 0 | 0.02 | 0.00 | 0 | 0.00 | 0.00 | 0.00 | 0.00 | 0 | 0.00 | 0.00 | 0 | 0 | 0.00 | 0.00 | 0.00 | 0.00 | 0.00 | 0.00 | 0 | 0 | 0 | 0 |
| **19** | 0 | 0 | 0 | 0 | 0 | 0 | 0 | 0 | 0 | 0 | 0 | 0 | 0 | 0 | 0 | 0 | 0 | 0 | 0 | 0 | 0 | 0 | 0 | 0 | 0 | 0 | 0 |
| **20** | 0.08 | 0.03 | 0 | 0 | 0.46 | 0.01 | 0 | 0.00 | 0.00 | 0 | 0.00 | 0.00 | 0 | 0.00 | 0.00 | 0 | 0 | 0.00 | 0.00 | 0.00 | 0.00 | 0.00 | 0.01 | 0 | 0 | 0 | 0 |
| **21** | 0 | 0.00 | 0 | 0 | 0 | 0.00 | 0 | 0 | 0 | 0 | 0 | 0 | 0 | 0 | 0 | 0 | 0 | 0 | 0 | 0 | 0 | 0 | 0 | 0 | 0 | 0 | 0 |
| **22** | 0 | 0.00 | 0 | 0 | 0 | 0 | 0 | 0 | 0 | 0 | 0 | 0 | 0 | 0 | 0 | 0 | 0 | 0 | 0 | 0 | 0 | 0 | 0 | 0 | 0 | 0 | 0 |
| **23** | 0 | 0.00 | 0 | 0 | 0 | 0.00 | 0 | 0 | 0 | 0 | 0 | 0 | 0 | 0 | 0 | 0 | 0 | 0 | 0 | 0 | 0 | 0 | 0 | 0 | 0 | 0 | 0 |
| **24** | 0.26 | 0.02 | 0.02 | 0.00 | 0 | 0.02 | 0.00 | 0 | 0.00 | 0.00 | 0.01 | 0.00 | 0.00 | 0.01 | 0.00 | 0 | 0 | 0.00 | 0.00 | 0.00 | 0.00 | 0.00 | 0.01 | 0 | 0 | 0.00 | 0 |
| **25** | 0.42 | 0.01 | 0 | 0 | 0 | 0.01 | 0 | 0 | 0.00 | 0 | 0.01 | 0.00 | 0 | 0.01 | 0.00 | 0 | 0 | 0.00 | 0.00 | 0.00 | 0.00 | 0.00 | 0.01 | 0 | 0 | 0 | 0 |
| **26** | 0.01 | 0.02 | 0.00 | 0 | 0 | 0.04 | 0.00 | 0 | 0.00 | 0 | 0.00 | 0.01 | 0 | 0.00 | 0.00 | 0 | 0 | 0.00 | 0.00 | 0.00 | 0.00 | 0.00 | 0.00 | 0 | 0 | 0 | 0 |
| **27** | 0 | 0.00 | 0 | 0 | 0 | 0 | 0 | 0 | 0 | 0 | 0 | 0 | 0 | 0 | 0 | 0 | 0 | 0 | 0 | 0 | 0 | 0 | 0 | 0 | 0 | 0 | 0 |
| **28** | 0.06 | 0.01 | 0.00 | 0 | 0 | 0.01 | 0.00 | 0 | 0.00 | 0 | 0.00 | 0.00 | 0 | 0.00 | 0.00 | 0 | 0 | 0.00 | 0.00 | 0.00 | 0.00 | 0.00 | 0.00 | 0 | 0 | 0 | 0 |

19: Frisian; 20: Henson; 21: Icelandic Horse; 22: Lipizzan; 23: Lusitano horse; 24: Other foreign race and riding horses; 25: Royal Dutch Sport Horse; 26: Shagya; 27: Spanish Purebred; 28: Trakehner; 29: Zangersheide; 30: Certified Pony Origin; 31: Connemara; 32: Dartmoor; 33: Fjord; 34: French Saddle Pony; 35: Haflinger; 36: Highland; 37: Landais; 38: New-Forest;

| **code** | **29** | **30** | **31** | **32** | **33** | **34** | **35** | **36** | **37** | **38** | **39** | **40** | **41** | **42** | **43** | **44** | **45** | **46** | **47** | **48** | **49** | **50** | **51** | **52** | **53** | **54** | **55** |
| --- | --- | --- | --- | --- | --- | --- | --- | --- | --- | --- | --- | --- | --- | --- | --- | --- | --- | --- | --- | --- | --- | --- | --- | --- | --- | --- | --- |
| **29** | 1.12 | 0.04 | 0.00 | 0 | 0 | 0.03 | 0 | 0 | 0.00 | 0 | 0.01 | 0.00 | 0 | 0.02 | 0.00 | 0 | 0 | 0.00 | 0.00 | 0.00 | 0.00 | 0.00 | 0.02 | 0 | 0 | 0 | 0 |
| **30** |  | 0.18 | 0.64 | 0.05 | 0.05 | 0.27 | 0.08 | 0.01 | 0.06 | 0.16 | 0.01 | 0.02 | 0.03 | 0.14 | 0.10 | 0.00 | 0.00 | 0.00 | 0.00 | 0.00 | 0.00 | 0.00 | 0.00 | 0 | 0.00 | 0.00 | 0 |
| **31** |  |  | 3.84 | 0 | 0 | 0.92 | 0 | 0 | 0.00 | 0 | 0.04 | 0.00 | 0 | 0.01 | 0.00 | 0 | 0 | 0.00 | 0.00 | 0.00 | 0 | 0 | 0 | 0 | 0 | 0 | 0 |
| **32** |  |  |  | 3.82 | 0 | 0.02 | 0 | 0 | 0 | 0 | 0 | 0 | 0 | 0.00 | 0 | 0 | 0 | 0 | 0 | 0 | 0 | 0 | 0 | 0 | 0 | 0 | 0 |
| **33** |  |  |  |  | 1.59 | 0.00 | 0 | 0 | 0 | 0 | 0 | 0 | 0 | 0 | 0 | 0 | 0 | 0 | 0 | 0.00 | 0 | 0 | 0 | 0 | 0 | 0 | 0 |
| **34** |  |  |  |  |  | 0.55 | 0.00 | 0.00 | 0.06 | 0.33 | 0.01 | 0.01 | 0.00 | 0.18 | 0.11 | 0 | 0 | 0.00 | 0.00 | 0.00 | 0.00 | 0.00 | 0.00 | 0 | 0 | 0 | 0 |
| **35** |  |  |  |  |  |  | 4.22 | 0 | 0.00 | 0 | 0.00 | 0.00 | 0 | 0.01 | 0 | 0 | 0 | 0.00 | 0 | 0.00 | 0 | 0 | 0 | 0 | 0 | 0 | 0 |
| **36** |  |  |  |  |  |  |  | 4.31 | 0 | 0 | 0 | 0 | 0 | 0 | 0 | 0 | 0 | 0 | 0 | 0 | 0 | 0 | 0 | 0 | 0 | 0 | 0 |
| **37** |  |  |  |  |  |  |  |  | 4.73 | 0 | 0.00 | 0.00 | 0 | 0.00 | 0.00 | 0 | 0 | 0.00 | 0.00 | 0.00 | 0 | 0 | 0.00 | 0 | 0 | 0 | 0 |
| **38** |  |  |  |  |  |  |  |  |  | 1.96 | 0.00 | 0.01 | 0 | 0.00 | 0.00 | 0 | 0 | 0 | 0 | 0.00 | 0 | 0 | 0 | 0 | 0 | 0 | 0 |
| **39** |  |  |  |  |  |  |  |  |  |  | 8.25 | 0.00 | 0 | 0.00 | 0.01 | 0 | 0 | 0.00 | 0.00 | 0.00 | 0.00 | 0.00 | 0.00 | 0 | 0 | 0 | 0 |
| **40** |  |  |  |  |  |  |  |  |  |  |  | 0.44 | 0 | 0.01 | 0.01 | 0 | 0 | 0.00 | 0.00 | 0.00 | 0.00 | 0.00 | 0.00 | 0 | 0 | 0 | 0 |
| **41** |  |  |  |  |  |  |  |  |  |  |  |  | 0.64 | 0.00 | 0 | 0 | 0 | 0 | 0 | 0.00 | 0 | 0 | 0 | 0 | 0 | 0 | 0 |
| **42** |  |  |  |  |  |  |  |  |  |  |  |  |  | 3.09 | 0.28 | 0.00 | 0.00 | 0.00 | 0.00 | 0.00 | 0.00 | 0.00 | 0.00 | 0 | 0 | 0.00 | 0.00 |
| **43** |  |  |  |  |  |  |  |  |  |  |  |  |  |  | 1.64 | 0 | 0 | 0 | 0 | 0.00 | 0 | 0 | 0.00 | 0 | 0 | 0 | 0 |
| **44** |  |  |  |  |  |  |  |  |  |  |  |  |  |  |  | 0.60 | 0.17 | 0 | 0.00 | 0.03 | 0.00 | 0.01 | 0 | 0.03 | 0.00 | 0.00 | 0.02 |
| **45** |  |  |  |  |  |  |  |  |  |  |  |  |  |  |  |  | 1.21 | 0 | 0.00 | 0.01 | 0.00 | 0.00 | 0 | 0.17 | 0.00 | 0.00 | 0.33 |
| **46** |  |  |  |  |  |  |  |  |  |  |  |  |  |  |  |  |  | 7.10 | 0 | 0.02 | 0 | 0 | 0.00 | 0 | 0.00 | 0 | 0.00 |
| **47** |  |  |  |  |  |  |  |  |  |  |  |  |  |  |  |  |  |  | 0.87 | 0.23 | 0.00 | 0.00 | 0.00 | 0.00 | 0.00 | 0.00 | 0.00 |
| **48** |  |  |  |  |  |  |  |  |  |  |  |  |  |  |  |  |  |  |  | 0.28 | 0.03 | 0.69 | 0.00 | 0.00 | 0.03 | 0.02 | 0.01 |
| **49** |  |  |  |  |  |  |  |  |  |  |  |  |  |  |  |  |  |  |  |  | 1.65 | 0.00 | 0.00 | 0 | 0.00 | 0.00 | 0.00 |
| **50** |  |  |  |  |  |  |  |  |  |  |  |  |  |  |  |  |  |  |  |  |  | 2.37 | 0.00 | 0 | 0.00 | 0.00 | 0.00 |
| **51** |  |  |  |  |  |  |  |  |  |  |  |  |  |  |  |  |  |  |  |  |  |  | 2.48 | 0 | 0 | 0 | 0 |
| **52** |  |  |  |  |  |  |  |  |  |  |  |  |  |  |  |  |  |  |  |  |  |  |  | 2.89 | 0 | 0 | 0.31 |
| **53** |  |  |  |  |  |  |  |  |  |  |  |  |  |  |  |  |  |  |  |  |  |  |  |  | 0.87 | 0.00 | 0.00 |
| **54** |  |  |  |  |  |  |  |  |  |  |  |  |  |  |  |  |  |  |  |  |  |  |  |  |  | 7.55 | 0.00 |
| **55** |  |  |  |  |  |  |  |  |  |  |  |  |  |  |  |  |  |  |  |  |  |  |  |  |  |  | 1.38 |

39: Other foreign pony; 40: Pottok; 41: Shetland; 42: Welsh Cob; 43: Welsh Pony; 44: Ardennais; 45: Auxois; 46: Boulonnais; 47: Breton; 48: Certified draught horse origin; 49: Cob Normand; 50: Comtois; 51: Franches-Montagnes; 52: Other foreign draught horse; 53: Percheron; 54: Poitevin Mulassier; 55: Trait du Nord
