# Supplementary material for: Genetic Structure and Gene Flows within Horses: A Genealogical Study at the French Population Scale
Source: PLoS One. 2013 Apr 22;8(4):e61544. doi: 10.1371/journal.pone.0061544 (PMC3632587; doi:10.1371/journal.pone.0061544)
Supplement: Table S4 — Founder origins of the race and riding breeds and groups of breeds (%). (DOCX) [file pone.0061544.s004.docx]

**Table S4. Founder origins of the race and riding breeds and groups of breeds (%).**

| Group | code | 1 | 2 | 3 | 4 | 5 | 6 | 7 | 8 | 9 | 10 | 11 | 12 | 13 |
| --- | --- | --- | --- | --- | --- | --- | --- | --- | --- | --- | --- | --- | --- | --- |
| American breeds | 1 | 95.7 | 0 | 0 | 0 | 0 | 0.6 | 3.4 | 0 | 0 | 0 | 0 | 0 | 0.3 |
| AQPS | 2 | 0 | 0 | 1.1 | 0.4 | 0.0 | 0.8 | 97.3 | 0.3 | 0 | 0 | 0.0 | 0 | 0.0 |
| Selle Français | 3 | 0.0 | 0 | 32.6 | 2.7 | 0.0 | 2.5 | 46.7 | 4.1 | 0 | 0.0 | 0.1 | 0.0 | 11.2 |
| Anglo-Arab | 4 | 0.0 | 0 | 3.4 | 13.1 | 0.1 | 26.7 | 54.4 | 0.6 | 0 | 0.0 | 0.0 | 0.0 | 1.7 |
| Half Bred Arab | 5 | 0.3 | 0 | 0.9 | 0.6 | 24.8 | 57.4 | 3.6 | 3.8 | 0.5 | 0.4 | 2.3 | 2.1 | 3.3 |
| Arab | 6 | 0 | 0 | 0 | 0 | 0 | 100.0 | 0 | 0 | 0 | 0 | 0 | 0 | 0 |
| Thoroughbred | 7 | 0 | 0 | 0.0 | 0 | 0 | 0 | 100.0 | 0 | 0 | 0 | 0 | 0 | 0 |
| French Trotter | 8 | 0 | 0 | 0 | 0 | 0 | 0 | 0.9 | 82.1 | 0 | 0 | 0 | 0 | 17.0 |
| Merens | 9 | 0 | 0 | 0 | 0 | 0 | 0 | 0 | 0.6 | 99.3 | 0 | 0 | 0.1 | 0.0 |
| Camargue | 10 | 0 | 0 | 0 | 0 | 0 | 0 | 0 | 0 | 0 | 100.0 | 0 | 0 | 0 |
| Draught horses | 11 | 0 | 0 | 0.0 | 0.0 | 0.0 | 0.0 | 0.1 | 0.1 | 0.1 | 0.0 | 99.6 | 0.0 | 0.1 |
| Pony | 12 | 0.0 | 0 | 0.7 | 0.4 | 0.5 | 7.0 | 2.0 | 0.7 | 0.1 | 0.1 | 0.1 | 87.5 | 1.0 |
| Other race and riding horses | 13 | 5.0 | 0 | 5.3 | 1.5 | 0.5 | 5.5 | 14.5 | 2.6 | 0.9 | 0.6 | 0.8 | 2.8 | 60.0 |

(0.0 correspond to values different from absolute zero).
