# Supplementary material for: Genetic Structure and Gene Flows within Horses: A Genealogical Study at the French Population Scale
Source: PLoS One. 2013 Apr 22;8(4):e61544. doi: 10.1371/journal.pone.0061544 (PMC3632587; doi:10.1371/journal.pone.0061544)
Supplement: Document S1 — Program source code. (DOCX) [file pone.0061544.s005.docx]

**Document S1. Program source code**

We used Pedig software (<http://www-sgqa.jouy.inra.fr/article.php3?id_article=110> ) in order to obtain coancestries among populations studied, coancestry rates, but also the genes origins of each population. Observing the quantity of data, two programs of the software has been modified, considering slight changes in FORTRAN 77 routines. The third program computes coancestry rate among populations, in order to correct difference in pedigree knowledge between populations.

Each program should be compiled in the same folder than pedig source files. Pedigree file should follow the Pedig format, with the five parameters in column.

- Pedig individual identification
- Pedig sire identification
- Pedig dam identification
- Birth year
- Sex (1: male, 2: female)
- Reference population (1: belong to reference population, 0: does not belong to reference population)
- Origin (a numerical value).

Note reference subpopulation size and number of origins analyzed are here limited to 1 000 000 and 60, respectively.

**PAR3** (page 2)

This program originally allows computing coancestry coefficients between two populations. Here the program is modified to compute average coancestry among a larger number of populations (origins), considering a given number of pairs, chosen by the user, randomly sampled among each origin.

**ETR** (page 22)

The aim this program is to study the probability of genes origins of a reference population, with several populations inside, considering different origins. Similarly to PAR3, it has been modified to taken into account a larger number of populations (origins).

**CoanRate**  (page 27)

The program is a combination of par3.exe and ngen.exe Pedig programs. It computes average coancestry rate using Cervantes et al. among a large number of populations (origins), considering a given number of pairs, chosen by the user, randomly sampled among each origin.

**Par3**

C******** computation of coancestries ***********

program coancestry

implicit none

include "blk.incl"

integer*4 i, j, k,s,m,p,ii,jj,kk,is,il,ne,eff,iopt(4),na,ie,ip,im

integer*4 je,nd,nlist(60),iad,iaf,ifail,ig,ig1,ig2,v,nrefpop

integer*4 nbreed, nrep,n

character*128 str,sti,stc,day,fmt

integer*4 lliste(1000000,60)

integer*4 father(nt),mother(nt),ped(2,nt),point(nt),refpop(nt)

integer*4 ord(nt),rord(nt),id(nt),ic(nt),ndes(nt),breedcode(nt)

integer*4 base(nt)

real*8 statis(106),x,ranf,anint,ftot,ptot

real*8 f(0:nt),l(nt),d(nt),fmoy(60)

real*8 pmoy(60,60)

logical ts,ts2(60,60),chy

include "format.incl"

nbreed=60

call fdate(day)

PRINT 990,day

print 8001

print 1001

read(5,*) str

print 1002,str

print 1003

print *,'no if no output file'

read (5,*) stc

print 1004,stc

write (*,*) 'enter number of repetitions desired for coancestries'

read(*,*) nrep

ts=.true.

if (stc.eq.'no') ts=.false.

do i=1,nbreed

do j=1,nbreed

ts2(i,j)=ts

end do

end do

c pedigree reading

n=0

chy=.false.

nbreed=0

open (1,file=str,form='FORMATTED')

1 read (1,*,end=2) i,j,k,s,s,m,p

n=n+1

if (jj.le.11) then

jj=jj+2000

chy=.true.

else if (jj.le.100) then

jj=jj+1900

chy=.true.

end if

if (i.ne.n) then

print 102,n,i

stop

end if

if (i.gt.nt.or.j.gt.nt.or.k.gt.nt) then

print 101

stop

end if

father(n)=j

mother(n)=k

refpop(n)=m

breedcode(n)=p

if (breedcode(n).gt.nbreed.and.refpop(n).ne.0) then

nbreed=breedcode(n)

end if

if (j.lt.0) father(n)=0

if (k.lt.0) mother(n)=0

goto 1

2 if (chy) print 103

print 900, n

close(1)

write (*,*) ' populations considered: ',nbreed

do i=1,nbreed

nlist(i)=0

do j=1,1000000

lliste(j,i)=0

end do

end do

do ig1=1,nbreed

do ig2=1,nbreed

pmoy(ig1,ig2)=0

end do

end do

c reference population recovery

nrefpop=0

do i=1,n

if (refpop(i).ne.0) then

nrefpop=nrefpop+1

base(nrefpop)=i

nlist(breedcode(i))=nlist(breedcode(i))+1

lliste(nlist(breedcode(i)),breedcode(i))=i

end if

end do

write(*,*) 'Reference population size',nrefpop

c write(*,*) 'Populations sizes',(nlist(j)," ",j=1,nbreed)

do ig=1,nbreed

do i=1, nlist(ig)

if (lliste(i,ig).gt.n) then

print 8512,lliste(i,ig)

stop

end if

end do

end do

do i=1, n

if (father(i).gt.n) then ; print 8513; stop; end if

if (mother(i).gt.n) then ; print 8514; stop; end if

end do

c *** numbering from the oldest to the youngest

call comp_d (n, father, mother,ped, ord, rord)

do ig=1,nbreed

do i=1, nlist(ig)

ndes(ord(lliste(i,ig)))=1

end do

end do

do i=1,n

point(i)=0

l(i)=0.

d(i)=0.

end do

call meuw(n, ped, f, d, l, point,ndes)

if (ts) then

open (3,file=stc,form='formatted')

ftot=0

do ig=1,nbreed

fmoy(ig)=0

do i=1, nlist(ig)

j=lliste(i,ig)

c if (ts2(ig,ig)) write (3,*) j,f(ord(j)),ig,ig,

fmoy(ig)=fmoy(ig)+f(ord(lliste(i,ig)))

ftot=ftot+f(ord(lliste(i,ig)))

end do

fmoy(ig)=fmoy(ig)/dfloat(nlist(ig))

end do

end if

8888 format (2i8,f7.4,2i2)

write (3,40) (ig1,ig1=1,nbreed)

write (3,41) (fmoy(ig),ig=1,nbreed)

write (3,*)'Average total inbreeding', ftot/nrefpop

40 format (///' Inbreeding '/

* ' ******** '/

* 60i12)

41 format (60f12.9)

n=n + 1

do i=1, n

point(i)=0

l(i)=0.

end do

do ig1=1,nbreed

do ig2=ig1,nbreed

do i=1,nrep

ip=ord(lliste(ranf()*nlist(ig1)+1,ig1))

ped(1,n)=ip

666 im=ord(lliste(ranf()*nlist(ig2)+1,ig2))

ped(2,n)=im

if (im.eq.ip) goto 666

call inbreed(n,ped,f,d,l,point)

pmoy(ig1,ig2)=pmoy(ig1,ig2)+f(n)

end do

pmoy(ig1,ig2)=pmoy(ig1,ig2)/dfloat(nrep)

pmoy(ig2,ig1)=pmoy(ig1,ig2)

end do

end do

write (3,42) (ig1,ig1=1,nbreed)

do ig1=1,nbreed

write(3,43) ig1,nlist(ig1),(pmoy(ig1,ig2),ig2=1,nbreed)

c 212 format (55f12.9)

end do

42 format (///' Coancestries '/

* ' ******** '/

* 'Breed N ',60i12)

43 format (i4,i8,60f12.9)

call fdate(day)

print *

PRINT 991,day

stop

end

subroutine comp_d (n,sire, dam,ped,ord,rord)

implicit none

integer*4 n,sire(*),dam(*),ped(2,*),nbit,k,i,j,ks,kd

integer*4 ord(*), rord(*)

include "format.incl"

nbit=0

do i=1, n

ord(i)=0

end do

k=0

do while (k.lt.n .and. nbit.le.20)

nbit=nbit + 1

do i=1, n

if (ord(i).eq.0) then

if (sire(i).le.0 .or. ord(sire(i)).ne.0) then

if (dam(i) .le.0 .or. ord( dam(i)).ne.0) then

k=k+1

ord(i)=k

rord(k)=i

end if

end if

end if

end do

end do

c Test there is no loop in this pedigree

j=0

if (k.ne.n) then

do i=1,n

if (ord(i).eq.0) then

j=j+1

if (j.lt.100) print '(3i10)',i,sire(i),dam(i)

sire(i)=0

dam(i)=0

k=k+1

ord(i)=k

rord(k)=i

end if

end do

end if

if (j.gt.0) print 900,j

do i=1, n

j=rord(i)

ped(1,i)=sire(j)

ped(2,i)=dam(j)

if (ped(1,i).gt.0) ped(1,i)=ord(sire(j))

if (ped(2,i).gt.0) ped(2,i)=ord(dam(j))

end do

DO i=1,n

if (i.le.ped(1,i) .or. i.le.ped(2,i)) then

print *,'Problem in coding pedigree'

print *,i, ped(1,i),ped(2,i)

stop

end if

ks=ped(1,i)

kd=ped(2,i)

ped(1,i)=max(ks,kd)

ped(2,i)=min(ks,kd)

end do

return

end

C*** Methode de Meuwissen

subroutine meuw(n, ped, f, d, l ,point,ndes)

implicit none

integer*4 n, ped(2,*), point(*),ndes(*),np,npar

integer*4 ninbr, i, j,k, ik, is, id, ks, kd

real*8 f(0:n), d(*), l(*),r, fi

ninbr=0

f(0)=-1.d0

DO i=1,n

point(i)=0

end do

DO i=1,n

if (ped(1,i).gt.0) ndes(ped(1,i))=ndes(ped(1,i))+1

if (ped(2,i).gt.0) ndes(ped(2,i))=ndes(ped(2,i))+1

end do

npar=0

do i=1, n

if (ndes(i).gt.0) npar=npar+1

end do

if (npar.eq.0) return

DO i=1,n

if (ndes(i).gt.0) then

is=ped(1,i)

id=ped(2,i)

ped(1,i)=max(is,id)

ped(2,i)=min(is,id)

d(i)=.5d0 - .25d0*(f(is)+f(id))

if (is.eq.0.or.id.eq.0) then

f(i)=0.d0

else

np=0

fi=-1.d0

l(i)=1.d0

j=i

do while(j.ne.0)

k=j

r=.5d0 * l(k)

ks=ped(1,k)

kd=ped(2,k)

if (ks.gt.0) then

l(ks)=l(ks) + r

do while(point(k).gt.ks)

k=point(k)

end do

if (ks.ne.point(k)) then

point(ks)=point(k)

point(k)=ks

end if

if (kd.gt.0) then

l(kd)=l(kd) + r

do while(point(k).gt.kd)

k=point(k)

end do

if (kd.ne.point(k)) then

point(kd)=point(k)

point(k)=kd

end if

end if

end if

fi=fi + l(j)*l(j)*d(j)

l(j)=0.d0

k=j

j=point(j)

point(k)=0

np=np+1

end do

f(i)=fi

if (fi.gt.0.000001d0) ninbr=ninbr + 1

end if

end if

end do

RETURN

END

C*** Methode de Meuwissen

subroutine inbreed(i, ped, f, d, l ,point)

implicit none

integer*4 ped(2,*), point(*)

integer*4 i, j,k, ik, is, id, ks, kd

real*8 f(0:i), d(*), l(*),r, fi

is=ped(1,i)

id=ped(2,i)

ped(1,i)=max(is,id)

ped(2,i)=min(is,id)

d(i)=.5d0 - .25d0*(f(is)+f(id))

if (is.eq.0.or.id.eq.0) then

f(i)=0.d0

return

end if

fi=-1.d0

l(i)=1.d0

j=i

do while(j.ne.0)

k=j

r=.5d0 * l(k)

ks=ped(1,k)

kd=ped(2,k)

if (ks.gt.0) then

l(ks)=l(ks) + r

do while(point(k).gt.ks)

k=point(k)

end do

if (ks.ne.point(k)) then

point(ks)=point(k)

point(k)=ks

end if

if (kd.gt.0) then

l(kd)=l(kd) + r

do while(point(k).gt.kd)

k=point(k)

end do

if (kd.ne.point(k)) then

point(kd)=point(k)

point(k)=kd

end if

end if

end if

fi=fi + l(j)*l(j)*d(j)

l(j)=0.d0

k=j

j=point(j)

point(k)=0

end do

f(i)=fi

RETURN

END

SUBROUTINE getcgn(g)

INTEGER g

C**********************************************************************

C

C SUBROUTINE GETCGN(G)

C Get GeNerator

C

C Returns in G the number of the current random number generator

C

C

C Arguments

C

C

C G <-- Number of the current random number generator (1..32)

C INTEGER G

C

C**********************************************************************

C

INTEGER curntg,numg

SAVE curntg

PARAMETER (numg=32)

DATA curntg/1/

C

g = curntg

RETURN

ENTRY setcgn(g)

C**********************************************************************

C

C SUBROUTINE SETCGN( G )

C Set GeNerator

C

C Sets the current generator to G. All references to a generat

C are to the current generator.

C

C

C Arguments

C

C

C G --> Number of the current random number generator (1..32)

C INTEGER G

C

C**********************************************************************

C

C Abort if generator number out of range

C

IF (.NOT. (g.LT.0.OR.g.GT.numg)) GO TO 10

WRITE (*,*) ' Generator number out of range in SETCGN:',

+ ' Legal range is 1 to ',numg,' -- ABORT!'

STOP ' Generator number out of range in SETCGN'

10 curntg = g

RETURN

END

INTEGER FUNCTION ignlgi()

C**********************************************************************

C

C INTEGER FUNCTION IGNLGI()

C GeNerate LarGe Integer

C

C Returns a random integer following a uniform distribution over

C (1, 2147483562) using the current generator.

C

C This is a transcription from Pascal to Fortran of routine

C Random from the paper

C

C L'Ecuyer, P. and Cote, S. "Implementing a Random Number Package

C with Splitting Facilities." ACM Transactions on Mathematical

C Software, 17:98-111 (1991)

C

C**********************************************************************

C .. Parameters ..

INTEGER numg

PARAMETER (numg=32)

C ..

C .. Scalars in Common ..

INTEGER a1,a1vw,a1w,a2,a2vw,a2w,m1,m2

C ..

C .. Arrays in Common ..

INTEGER cg1(numg),cg2(numg),ig1(numg),ig2(numg),lg1(numg),

+ lg2(numg)

LOGICAL qanti(numg)

C ..

C .. Local Scalars ..

INTEGER curntg,k,s1,s2,z

LOGICAL qqssd

C ..

C .. External Functions ..

LOGICAL qrgnin

EXTERNAL qrgnin

C ..

C .. External Subroutines ..

EXTERNAL getcgn,inrgcm,rgnqsd,setall

C ..

C .. Common blocks ..

COMMON /globe/m1,m2,a1,a2,a1w,a2w,a1vw,a2vw,ig1,ig2,lg1,lg2,cg1,

+ cg2,qanti

C ..

C .. Save statement ..

SAVE /globe/

C ..

C .. Executable Statements ..

C

C IF THE RANDOM NUMBER PACKAGE HAS NOT BEEN INITIALIZED YET, DO SO.

C IT CAN BE INITIALIZED IN ONE OF TWO WAYS : 1) THE FIRST CALL TO

C THIS ROUTINE 2) A CALL TO SETALL.

C

IF (.NOT. (qrgnin())) CALL inrgcm()

CALL rgnqsd(qqssd)

IF (.NOT. (qqssd)) CALL setall(1234567890,123456789)

C

C Get Current Generator

C

CALL getcgn(curntg)

s1 = cg1(curntg)

s2 = cg2(curntg)

k = s1/53668

s1 = a1* (s1-k*53668) - k*12211

IF (s1.LT.0) s1 = s1 + m1

k = s2/52774

s2 = a2* (s2-k*52774) - k*3791

IF (s2.LT.0) s2 = s2 + m2

cg1(curntg) = s1

cg2(curntg) = s2

z = s1 - s2

IF (z.LT.1) z = z + m1 - 1

IF (qanti(curntg)) z = m1 - z

ignlgi = z

RETURN

END

SUBROUTINE initgn(isdtyp)

C**********************************************************************

C

C SUBROUTINE INITGN(ISDTYP)

C INIT-ialize current G-e-N-erator

C

C Reinitializes the state of the current generator

C

C This is a transcription from Pascal to Fortran of routine

C Init_Generator from the paper

C

C L'Ecuyer, P. and Cote, S. "Implementing a Random Number Package

C with Splitting Facilities." ACM Transactions on Mathematical

C Software, 17:98-111 (1991)

C

C

C Arguments

C

C

C ISDTYP -> The state to which the generator is to be set

C

C ISDTYP = -1 => sets the seeds to their initial value

C ISDTYP = 0 => sets the seeds to the first value of

C the current block

C ISDTYP = 1 => sets the seeds to the first value of

C the next block

C

C INTEGER ISDTYP

C

C**********************************************************************

C .. Parameters ..

INTEGER numg

PARAMETER (numg=32)

C ..

C .. Scalar Arguments ..

INTEGER isdtyp

C ..

C .. Scalars in Common ..

INTEGER a1,a1vw,a1w,a2,a2vw,a2w,m1,m2

C ..

C .. Arrays in Common ..

INTEGER cg1(numg),cg2(numg),ig1(numg),ig2(numg),lg1(numg),

+ lg2(numg)

LOGICAL qanti(numg)

C ..

C .. Local Scalars ..

INTEGER g

C ..

C .. External Functions ..

LOGICAL qrgnin

INTEGER mltmod

EXTERNAL qrgnin,mltmod

C ..

C .. External Subroutines ..

EXTERNAL getcgn

C ..

C .. Common blocks ..

COMMON /globe/m1,m2,a1,a2,a1w,a2w,a1vw,a2vw,ig1,ig2,lg1,lg2,cg1,

+ cg2,qanti

C ..

C .. Save statement ..

SAVE /globe/

C ..

C .. Executable Statements ..

C Abort unless random number generator initialized

IF (qrgnin()) GO TO 10

WRITE (*,*) ' INITGN called before random number generator ',

+ ' initialized -- abort!'

STOP ' INITGN called before random number generator initialized'

10 CALL getcgn(g)

IF ((-1).NE. (isdtyp)) GO TO 20

lg1(g) = ig1(g)

lg2(g) = ig2(g)

GO TO 50

20 IF ((0).NE. (isdtyp)) GO TO 30

CONTINUE

GO TO 50

C do nothing

30 IF ((1).NE. (isdtyp)) GO TO 40

lg1(g) = mltmod(a1w,lg1(g),m1)

lg2(g) = mltmod(a2w,lg2(g),m2)

GO TO 50

40 STOP 'ISDTYP NOT IN RANGE'

50 cg1(g) = lg1(g)

cg2(g) = lg2(g)

RETURN

END

SUBROUTINE inrgcm()

C**********************************************************************

C

C SUBROUTINE INRGCM()

C INitialize Random number Generator CoMmon

C

C

C Function

C

C

C Initializes common area for random number generator. This saves

C the nuisance of a BLOCK DATA routine and the difficulty of

C assuring that the routine is loaded with the other routines.

C

C**********************************************************************

C .. Parameters ..

INTEGER numg

PARAMETER (numg=32)

C ..

C .. Scalars in Common ..

INTEGER a1,a1vw,a1w,a2,a2vw,a2w,m1,m2

C ..

C .. Arrays in Common ..

INTEGER cg1(numg),cg2(numg),ig1(numg),ig2(numg),lg1(numg),

+ lg2(numg)

LOGICAL qanti(numg)

C ..

C .. Local Scalars ..

INTEGER i

LOGICAL qdum

C ..

C .. External Functions ..

LOGICAL qrgnsn

EXTERNAL qrgnsn

C ..

C .. Common blocks ..

COMMON /globe/m1,m2,a1,a2,a1w,a2w,a1vw,a2vw,ig1,ig2,lg1,lg2,cg1,

+ cg2,qanti

C ..

C .. Save statement ..

SAVE /globe/

C ..

C .. Executable Statements ..

C V=20; W=30;

C

C A1W = MOD(A1**(2**W),M1) A2W = MOD(A2**(2**W),M2)

C A1VW = MOD(A1**(2**(V+W)),M1) A2VW = MOD(A2**(2**(V+W)),M2)

C

C If V or W is changed A1W, A2W, A1VW, and A2VW need to be recomputed.

C An efficient way to precompute a**(2*j) MOD m is to start with

C a and square it j times modulo m using the function MLTMOD.

C

m1 = 2147483563

m2 = 2147483399

a1 = 40014

a2 = 40692

a1w = 1033780774

a2w = 1494757890

a1vw = 2082007225

a2vw = 784306273

DO 10,i = 1,numg

qanti(i) = .FALSE.

10 CONTINUE

C

C Tell the world that common has been initialized

C

qdum = qrgnsn(.TRUE.)

RETURN

END

INTEGER FUNCTION mltmod(a,s,m)

C**********************************************************************

C

C INTEGER FUNCTION MLTMOD(A,S,M)

C

C Returns (A*S) MOD M

C

C This is a transcription from Pascal to Fortran of routine

C MULtMod_Decompos from the paper

C

C L'Ecuyer, P. and Cote, S. "Implementing a Random Number Package

C with Splitting Facilities." ACM Transactions on Mathematical

C Software, 17:98-111 (1991)

C

C

C Arguments

C

C

C A, S, M -->

C INTEGER A,S,M

C

C**********************************************************************

C .. Parameters ..

INTEGER h

PARAMETER (h=32768)

C ..

C .. Scalar Arguments ..

INTEGER a,m,s

C ..

C .. Local Scalars ..

INTEGER a0,a1,k,p,q,qh,rh

C ..

C .. Executable Statements ..

C

C H = 2**((b-2)/2) where b = 32 because we are using a 32 bit

C machine. On a different machine recompute H

C

IF (.NOT. (a.LE.0.OR.a.GE.m.OR.s.LE.0.OR.s.GE.m)) GO TO 10

WRITE (*,*) ' A, M, S out of order in MLTMOD - ABORT!'

WRITE (*,*) ' A = ',a,' S = ',s,' M = ',m

WRITE (*,*) ' MLTMOD requires: 0 < A < M; 0 < S < M'

STOP ' A, M, S out of order in MLTMOD - ABORT!'

10 IF (.NOT. (a.LT.h)) GO TO 20

a0 = a

p = 0

GO TO 120

20 a1 = a/h

a0 = a - h*a1

qh = m/h

rh = m - h*qh

IF (.NOT. (a1.GE.h)) GO TO 50

a1 = a1 - h

k = s/qh

p = h* (s-k*qh) - k*rh

30 IF (.NOT. (p.LT.0)) GO TO 40

p = p + m

GO TO 30

40 GO TO 60

50 p = 0

C

C P = (A2*S*H)MOD M

C

60 IF (.NOT. (a1.NE.0)) GO TO 90

q = m/a1

k = s/q

p = p - k* (m-a1*q)

IF (p.GT.0) p = p - m

p = p + a1* (s-k*q)

70 IF (.NOT. (p.LT.0)) GO TO 80

p = p + m

GO TO 70

80 CONTINUE

90 k = p/qh

C

C P = ((A2*H + A1)*S)MOD M

C

p = h* (p-k*qh) - k*rh

100 IF (.NOT. (p.LT.0)) GO TO 110

p = p + m

GO TO 100

110 CONTINUE

120 IF (.NOT. (a0.NE.0)) GO TO 150

C

C P = ((A2*H + A1)*H*S)MOD M

C

q = m/a0

k = s/q

p = p - k* (m-a0*q)

IF (p.GT.0) p = p - m

p = p + a0* (s-k*q)

130 IF (.NOT. (p.LT.0)) GO TO 140

p = p + m

GO TO 130

140 CONTINUE

150 mltmod = p

C

RETURN

END

LOGICAL FUNCTION qrgnin()

C**********************************************************************

C

C LOGICAL FUNCTION QRGNIN()

C Q Random GeNerators INitialized?

C

C A trivial routine to determine whether or not the random

C number generator has been initialized. Returns .TRUE. if

C it has, else .FALSE.

C

C**********************************************************************

C .. Scalar Arguments ..

LOGICAL qvalue

C ..

C .. Local Scalars ..

LOGICAL qinit

C ..

C .. Entry Points ..

LOGICAL qrgnsn

C ..

C .. Save statement ..

SAVE qinit

C ..

C .. Data statements ..

DATA qinit/.FALSE./

C ..

C .. Executable Statements ..

qrgnin = qinit

RETURN

ENTRY qrgnsn(qvalue)

C**********************************************************************

C

C LOGICAL FUNCTION QRGNSN( QVALUE )

C Q Random GeNerators Set whether iNitialized

C

C Sets state of whether random number generator is initialized

C to QVALUE.

C

C This routine is actually an entry in QRGNIN, hence it is a

C logical function. It returns the (meaningless) value .TRUE.

C

C**********************************************************************

qinit = qvalue

qrgnsn = .TRUE.

RETURN

END

REAL FUNCTION ranf()

C**********************************************************************

C

C REAL FUNCTION RANF()

C RANDom number generator as a Function

C

C Returns a random floating point number from a uniform distribution

C over 0 - 1 (endpoints of this interval are not returned) using the

C current generator

C

C This is a transcription from Pascal to Fortran of routine

C Uniform_01 from the paper

C

C L'Ecuyer, P. and Cote, S. "Implementing a Random Number Package

C with Splitting Facilities." ACM Transactions on Mathematical

C Software, 17:98-111 (1991)

C

C**********************************************************************

C .. External Functions ..

INTEGER ignlgi

EXTERNAL ignlgi

C ..

C .. Executable Statements ..

C

C 4.656613057E-10 is 1/M1 M1 is set in a data statement in IGNLGI

C and is currently 2147483563. If M1 changes, change this also.

C

ranf = ignlgi()*4.656613057E-10

RETURN

END

SUBROUTINE setall(iseed1,iseed2)

C**********************************************************************

C

C SUBROUTINE SETALL(ISEED1,ISEED2)

C SET ALL random number generators

C

C Sets the initial seed of generator 1 to ISEED1 and ISEED2. The

C initial seeds of the other generators are set accordingly, and

C all generators states are set to these seeds.

C

C This is a transcription from Pascal to Fortran of routine

C Set_Initial_Seed from the paper

C

C L'Ecuyer, P. and Cote, S. "Implementing a Random Number Package

C with Splitting Facilities." ACM Transactions on Mathematical

C Software, 17:98-111 (1991)

C

C

C Arguments

C

C

C ISEED1 -> First of two integer seeds

C INTEGER ISEED1

C

C ISEED2 -> Second of two integer seeds

C INTEGER ISEED1

C

C**********************************************************************

C .. Parameters ..

INTEGER numg

PARAMETER (numg=32)

C ..

C .. Scalar Arguments ..

INTEGER iseed1,iseed2

LOGICAL qssd

C ..

C .. Scalars in Common ..

INTEGER a1,a1vw,a1w,a2,a2vw,a2w,m1,m2

C ..

C .. Arrays in Common ..

INTEGER cg1(numg),cg2(numg),ig1(numg),ig2(numg),lg1(numg),

+ lg2(numg)

LOGICAL qanti(numg)

C ..

C .. Local Scalars ..

INTEGER g,ocgn

LOGICAL qqssd

C ..

C .. External Functions ..

INTEGER mltmod

LOGICAL qrgnin

EXTERNAL mltmod,qrgnin

C ..

C .. External Subroutines ..

EXTERNAL getcgn,initgn,inrgcm,setcgn

C ..

C .. Common blocks ..

COMMON /globe/m1,m2,a1,a2,a1w,a2w,a1vw,a2vw,ig1,ig2,lg1,lg2,cg1,

+ cg2,qanti

C ..

C .. Save statement ..

SAVE /globe/,qqssd

C ..

C .. Data statements ..

DATA qqssd/.FALSE./

C ..

C .. Executable Statements ..

C

C TELL IGNLGI, THE ACTUAL NUMBER GENERATOR, THAT THIS ROUTINE

C HAS BEEN CALLED.

C

qqssd = .TRUE.

CALL getcgn(ocgn)

C

C Initialize Common Block if Necessary

C

IF (.NOT. (qrgnin())) CALL inrgcm()

ig1(1) = iseed1

ig2(1) = iseed2

CALL initgn(-1)

DO 10,g = 2,numg

ig1(g) = mltmod(a1vw,ig1(g-1),m1)

ig2(g) = mltmod(a2vw,ig2(g-1),m2)

CALL setcgn(g)

CALL initgn(-1)

10 CONTINUE

CALL setcgn(ocgn)

RETURN

ENTRY rgnqsd(qssd)

C**********************************************************************

C

C SUBROUTINE RGNQSD

C Random Number Generator Query SeeD set?

C

C Returns (LOGICAL) QSSD as .TRUE. if SETALL has been invoked,

C otherwise returns .FALSE.

C

C**********************************************************************

qssd = qqssd

RETURN

END

**Etr**

C******** probability of genes origin ***********

program etr

include "blk.incl"

integer prc(nt), mrc(nt), orig(nt),refpop(nt)

integer*2 ann(nt), sex(nt)

integer*2 tabp(ng,1:ng),tabm(ng,1:ng)

integer i,j,k,l,nbit,ip,im,ngg, nl,jj,kk,ngre,ir,p

character*128 str,day,sor

real*8 x,y,moy(ng,1:ng),prob(0:ng,nt)

integer eff(ng)

logical test,chy

c real*4 t1, t2, gtimer,sum

call fdate(day)

PRINT 990,day

print 1000

print 1001

read(5,*) str

print 1002,str

print 1003

read(5,*) sor

print 1004,sor

c reading

open (1,file=str,form='FORMATTED')

nl=0

ngg=0

chy=.false.

10 read (1,*,end=20) i,ip,im,l,s,p,k

NL=NL+1

if (l.lt.11) then

l=l+2000

chy=.true.

else if (l.lt.100) then

l=l+1900

chy=.true.

end if

if (i.ne.nl) then

print 102,nl,i

stop

end if

if (i.gt.nt.or.ip.gt.nt.or.im.gt.nt) then

print 101

stop

end if

prc(nl)=ip

if (ip.lt.0) prc(nl)=0

mrc(nl)=im

if (im.lt.0) mrc(nl)=0

orig(nl)=k

ann(nl)=l

refpop(nl)=p

if (l.lt.pran) ann(nl)=pran

if (l.gt.dean) ann(nl)=dean

if (k.gt.ngg) ngg=k

GOTO 10

20 if (chy) print 103

print 900 , nl

print 1101, ngg

close(1)

do i=1,ngg

do j=1,ngg

tabp(j,i)=0

tabm(j,i)=0

end do

end do

j=0

open (9,file=sor,form='formatted')

c initialisations

k=0

do i=1, nl

prob(0,i)=-1

if (prc(i).eq.0 .and. mrc(i).eq.0) then

prob(0,i)=0

k=k+1

do j=1,ngg

prob(j,i)=0

end do

prob(orig(i),i)=1000

end if

end do

c construction de prob

c t1=gtimer()

nbit=0

test=.true.

do while(test .and. nbit.le.20)

nbit=nbit+1

j=0

test=.false.

do i=1, nl

if (prob(0,i).eq.-1) then

ip=prc(i)

im=mrc(i)

if (ip.gt.0 .and. im.gt.0) then

sum=0

if (prob(0,ip).ge.0 .and. prob(0,im).ge.0) then

do ig=1, ngg

prob(ig,i)=(prob(ig,ip)+prob(ig,im))/dfloat(2)

sum=sum+((prob(ig,ip)+prob(ig,im))/dfloat(2))

end do

prob(0,i)=0

k=k+1

end if

else if (ip.gt.0) then

if (prob(0,ip).ge.0) then

do ig=1, ngg

prob(ig,i)=prob(ig,ip)/dfloat(2)

sum=sum+(prob(ig,ip)/dfloat(2))

end do

prob(orig(i),i)=prob(orig(i),i)+500

sum=sum+500

prob(0,i)=0

k=k+1

end if

else if (im.gt.0) then

if (prob(0,im).ge.0) then

do ig=1, ngg

prob(ig,i)=prob(ig,im)/dfloat(2)

sum=sum+(prob(ig,im)/dfloat(2))

end do

prob(orig(i),i)=prob(orig(i),i)+500

sum=sum+500

prob(0,i)=0

k=k+1

end if

end if

end if

end do

if (k.lt.nl) test=.true.

end do

if (sum.le.999) then

write (*,*) i, sum,(prob(ig,i)," ",ig=1,ngg)

end if

c Test there is no loop in the pedigree

j=0

if (k.ne.nl) then

do i=1,nl

if (prob(0,i).lt.0) then

j=j+1

if (j.lt.100) print '(3i10)',i,prc(i),mrc(i)

prc(i)=0

mrc(i)=0

prob(0,i)=0

end if

end do

end if

if (j.gt.0) print 1104, j

c stat

do ir=1,ngg

eff(ir)=0.d0

do ig=1, ngg

moy(ig,ir)=0.d0

end do

end do

do i=1, nl

if (refpop(i).gt.0) then

ir=orig(i)

if (prc(i).ge.0 .and. mrc(i).ge.0) then

eff(ir)=eff(ir)+1

do ig=1, ngg

moy(ig,ir)=moy(ig,ir)+prob(ig,i)

end do

end if

end if

end do

write (9,666) (ig,ig=1,ngg)

do i=1,ngg

do ig=1, ngg

moy(ig,i)=moy(ig,i)/dfloat(eff(i))

end do

write (9,40) i,eff(i),(moy(ig,i),ig=1,ngg)

666 format (///' Origins '/

* ' ******** '/

* 'Breed N ',60i8)

40 format (i4,i8,60f8.2)

end do

c print *, 'Temps cpu : ',t2-t1

call fdate(day)

PRINT 991,day

include "format.incl"

stop

END

**CoanRate**

C******** computation of coancestry rates ***********

program coancestry

implicit none

include "blk.incl"

integer*4 i, j, k,s,m,p,ii,jj,kk,is,il,ne,eff,iopt(4),na,ie,ip,im

integer*4 je,nd,nlist(60),iad,iaf,ifail,ig,ig1,ig2,v,nrefpop

integer*4 nbreed, nrep,n,nanc(nt),ip1,im1

character*128 str,sti,stc,day,fmt

integer*4 lliste(1000000,60)

integer*4 father(nt),mother(nt),ped(2,nt),point(nt),refpop(nt)

integer*4 ord(nt),rord(nt),id(nt),ic(nt),ndes(nt),breedcode(nt)

integer*4 base(nt)

real*8 statis(106),x,ranf,anint,ftot,ptot,tab(ng,nt),eqg(nt)

real*8 f(0:nt),l(nt),d(nt),fmoy(60)

real*8 pmoy(60,60)

logical ts,ts2(60,60),chy,test,t

integer*4 ngene(nt),eff2(0:ng),nbit

integer nl

include "format.incl"

nbreed=60

call fdate(day)

PRINT 990,day

print 8001

print 1001

read(5,*) str

print 1002,str

print 1003

print *,'no if no output file'

read (5,*) stc

print 1004,stc

write (*,*) 'enter number of repetitions desired for coancestries'

read(*,*) nrep

ts=.true.

if (stc.eq.'no') ts=.false.

do i=1,nbreed

do j=1,nbreed

ts2(i,j)=ts

end do

end do

c pedigree reading

n=0

chy=.false.

nbreed=0

open (1,file=str,form='FORMATTED')

1 read (1,*,end=2) i,j,k,s,s,m,p

n=n+1

if (jj.le.11) then

jj=jj+2000

chy=.true.

else if (jj.le.100) then

jj=jj+1900

chy=.true.

end if

if (i.ne.n) then

print 102,n,i

stop

end if

if (i.gt.nt.or.j.gt.nt.or.k.gt.nt) then

print 101

stop

end if

father(n)=j

mother(n)=k

refpop(n)=m

breedcode(n)=p

if (breedcode(n).gt.nbreed.and.refpop(n).ne.0) then

nbreed=breedcode(n)

end if

if (j.lt.0) father(n)=0

if (k.lt.0) mother(n)=0

goto 1

2 if (chy) print 103

print 900, n

close(1)

nl=n

write (*,*) ' populations considered: ',nbreed

do i=1,nbreed

nlist(i)=0

do j=1,1000000

lliste(j,i)=0

end do

end do

do ig1=1,nbreed

do ig2=1,nbreed

pmoy(ig1,ig2)=0

end do

end do

c computation of pedigree knowledge

do i=1, nl

nanc(i)=0

do j=1, ng

tab(j,i)=0.

end do

end do

do j=0, ng

eff2(j)=0

end do

c pedigree checking

k=0

do i=1, nl

ngene(i)=-1

if (father(i).eq.0 .and. mother(i).eq.0) then

ngene(i)=0

k=k+1

end if

end do

nbit=0

test=.true.

c t1 = gtimer()

do while(test .and. nbit.le.50)

nbit=nbit+1

j=0

test=.false.

do i=1, nl

if (ngene(i).lt.0) then

ip=father(i)

im=mother(i)

t=.true.

if (ip.ne.0 .and. ngene(ip).lt.0) t=.false.

if (im.ne.0 .and. ngene(im).lt.0) t=.false.

if (t) then

if (ip.eq.0) then

ii=0

else

ii=ngene(ip)

end if

if (im.ne.0) ii=max(ii, ngene(im))

ngene(i) = ii+1

j=j+1

if (ip.gt.0) then

nanc(i)=nanc(i)+nanc(ip)+1

call add(i,ip,tab,nt,ng)

end if

if (im.gt.0) then

nanc(i)=nanc(i)+nanc(im)+1

call add(i,im,tab,nt,ng)

end if

else

test=.true.

end if

end if

end do

k=k+j

c print 1000,nbit,k,j,nl-k

end do

j=0

if (k.ne.nl) then

do i=1,nl

if (ngene(i).lt.0) then

j=j+1

if (j.lt.100) print '(3i10)',i,father(i),mother(i)

end if

end do

end if

if (j.gt.0) print 1104, j

c t2=gtimer()

c print *, 'Temps cpu : ', t2-t1

do i=1, nl

ii=ngene(i)

eff2(ii)=eff2(ii)+1

end do

c number of generation known computation

do i=1, nl

eqg(i)=0

do j=1,ng

eqg(i)=eqg(i)+tab(j,i)

end do

end do

c reference population recovery

nrefpop=0

do i=1,n

if (refpop(i).ne.0) then

nrefpop=nrefpop+1

base(nrefpop)=i

nlist(breedcode(i))=nlist(breedcode(i))+1

lliste(nlist(breedcode(i)),breedcode(i))=i

end if

end do

write(*,*) 'Reference population size',nrefpop

c write(*,*) 'Populations sizes',(nlist(j)," ",j=1,nbreed)

do ig=1,nbreed

do i=1, nlist(ig)

if (lliste(i,ig).gt.n) then

print 8512,lliste(i,ig)

stop

end if

end do

end do

do i=1, n

if (father(i).gt.n) then ; print 8513; stop; end if

if (mother(i).gt.n) then ; print 8514; stop; end if

end do

c *** numbering from the oldest to the youngest

call comp_d (n, father, mother,ped, ord, rord)

do ig=1,nbreed

do i=1, nlist(ig)

ndes(ord(lliste(i,ig)))=1

end do

end do

do i=1,n

point(i)=0

l(i)=0.

d(i)=0.

end do

call meuw(n, ped, f, d, l, point,ndes)

if (ts) then

open (3,file=stc,form='formatted')

ftot=0

do ig=1,nbreed

fmoy(ig)=0

do i=1, nlist(ig)

j=lliste(i,ig)

c if (ts2(ig,ig)) write (3,*) j,f(ord(j)),ig,ig,

c fmoy(ig)=fmoy(ig)+f(ord(lliste(i,ig)))

fmoy(ig)=fmoy(ig)+f(ord(lliste(i,ig)))

ftot=ftot+f(ord(lliste(i,ig)))

end do

fmoy(ig)=fmoy(ig)/dfloat(nlist(ig))

end do

end if

n=n + 1

do i=1, n

point(i)=0

l(i)=0.

end do

do ig1=1,nbreed

do ig2=ig1,nbreed

do i=1,nrep

x=ranf()

ip=ord(lliste(x*nlist(ig1)+1,ig1))

ip1=lliste(x*nlist(ig1)+1,ig1)

ped(1,n)=ip

666 x=ranf()

im=ord(lliste(x*nlist(ig2)+1,ig2))

im1=lliste(x*nlist(ig2)+1,ig2)

ped(2,n)=im

if (im.eq.ip) goto 666

call inbreed(n,ped,f,d,l,point)

if (i.eq.1) write(*,*) ig1,ig2,ip1,im1

* ,eqg(ip1),eqg(im1),f(n),1-(1-f(n))**(2/(eqg(ip1)+eqg(im1)) )

pmoy(ig1,ig2)=pmoy(ig1,ig2)+1-(1-f(n))**(2/(eqg(ip1)+eqg(im1)) )

end do

pmoy(ig1,ig2)=pmoy(ig1,ig2)/dfloat(nrep)

pmoy(ig2,ig1)=pmoy(ig1,ig2)

end do

end do

write (3,42) (ig1,ig1=1,nbreed)

do ig1=1,nbreed

write(3,43) ig1,nlist(ig1),(pmoy(ig1,ig2),ig2=1,nbreed)

c 212 format (55f12.9)

end do

42 format (///' Coancestry rates '/

* ' ******** '/

* 'Breed N ',60i12)

43 format (i4,i8,60f12.9)

call fdate(day)

print *

PRINT 991,day

stop

end

subroutine add(i,ip,tab,nt,ng)

integer*4 i,ip,nt,ng,j,k,ng1

real*8 tab(ng,nt)

ng1=ng-1

tab(1,i)=tab(1,i) + .5

if (tab(ng,ip).gt.0.) stop 'accroitre le parametre ng'

do j=1, ng1

if (tab(j,ip).eq.0.) return

k=j+1

tab(k,i) = tab(k,i) + tab(j,ip)*.5

end do

return

end

subroutine comp_d (n,sire, dam,ped,ord,rord)

implicit none

integer*4 n,sire(*),dam(*),ped(2,*),nbit,k,i,j,ks,kd

integer*4 ord(*), rord(*)

include "format.incl"

nbit=0

do i=1, n

ord(i)=0

end do

k=0

do while (k.lt.n .and. nbit.le.20)

nbit=nbit + 1

do i=1, n

if (ord(i).eq.0) then

if (sire(i).le.0 .or. ord(sire(i)).ne.0) then

if (dam(i) .le.0 .or. ord( dam(i)).ne.0) then

k=k+1

ord(i)=k

rord(k)=i

end if

end if

end if

end do

end do

c Test there is no loop in this pedigree

j=0

if (k.ne.n) then

do i=1,n

if (ord(i).eq.0) then

j=j+1

if (j.lt.100) print '(3i10)',i,sire(i),dam(i)

sire(i)=0

dam(i)=0

k=k+1

ord(i)=k

rord(k)=i

end if

end do

end if

if (j.gt.0) print 900,j

do i=1, n

j=rord(i)

ped(1,i)=sire(j)

ped(2,i)=dam(j)

if (ped(1,i).gt.0) ped(1,i)=ord(sire(j))

if (ped(2,i).gt.0) ped(2,i)=ord(dam(j))

end do

DO i=1,n

if (i.le.ped(1,i) .or. i.le.ped(2,i)) then

print *,'Problem in coding pedigree'

print *,i, ped(1,i),ped(2,i)

stop

end if

ks=ped(1,i)

kd=ped(2,i)

ped(1,i)=max(ks,kd)

ped(2,i)=min(ks,kd)

end do

return

end

C*** Methode de Meuwissen

subroutine meuw(n, ped, f, d, l ,point,ndes)

implicit none

integer*4 n, ped(2,*), point(*),ndes(*),np,npar

integer*4 ninbr, i, j,k, ik, is, id, ks, kd

real*8 f(0:n), d(*), l(*),r, fi

ninbr=0

f(0)=-1.d0

DO i=1,n

point(i)=0

end do

DO i=1,n

if (ped(1,i).gt.0) ndes(ped(1,i))=ndes(ped(1,i))+1

if (ped(2,i).gt.0) ndes(ped(2,i))=ndes(ped(2,i))+1

end do

npar=0

do i=1, n

if (ndes(i).gt.0) npar=npar+1

end do

if (npar.eq.0) return

DO i=1,n

if (ndes(i).gt.0) then

is=ped(1,i)

id=ped(2,i)

ped(1,i)=max(is,id)

ped(2,i)=min(is,id)

d(i)=.5d0 - .25d0*(f(is)+f(id))

if (is.eq.0.or.id.eq.0) then

f(i)=0.d0

else

np=0

fi=-1.d0

l(i)=1.d0

j=i

do while(j.ne.0)

k=j

r=.5d0 * l(k)

ks=ped(1,k)

kd=ped(2,k)

if (ks.gt.0) then

l(ks)=l(ks) + r

do while(point(k).gt.ks)

k=point(k)

end do

if (ks.ne.point(k)) then

point(ks)=point(k)

point(k)=ks

end if

if (kd.gt.0) then

l(kd)=l(kd) + r

do while(point(k).gt.kd)

k=point(k)

end do

if (kd.ne.point(k)) then

point(kd)=point(k)

point(k)=kd

end if

end if

end if

fi=fi + l(j)*l(j)*d(j)

l(j)=0.d0

k=j

j=point(j)

point(k)=0

np=np+1

end do

f(i)=fi

if (fi.gt.0.000001d0) ninbr=ninbr + 1

end if

end if

end do

RETURN

END

C*** Methode de Meuwissen

subroutine inbreed(i, ped, f, d, l ,point)

implicit none

integer*4 ped(2,*), point(*)

integer*4 i, j,k, ik, is, id, ks, kd

real*8 f(0:i), d(*), l(*),r, fi

is=ped(1,i)

id=ped(2,i)

ped(1,i)=max(is,id)

ped(2,i)=min(is,id)

d(i)=.5d0 - .25d0*(f(is)+f(id))

if (is.eq.0.or.id.eq.0) then

f(i)=0.d0

return

end if

fi=-1.d0

l(i)=1.d0

j=i

do while(j.ne.0)

k=j

r=.5d0 * l(k)

ks=ped(1,k)

kd=ped(2,k)

if (ks.gt.0) then

l(ks)=l(ks) + r

do while(point(k).gt.ks)

k=point(k)

end do

if (ks.ne.point(k)) then

point(ks)=point(k)

point(k)=ks

end if

if (kd.gt.0) then

l(kd)=l(kd) + r

do while(point(k).gt.kd)

k=point(k)

end do

if (kd.ne.point(k)) then

point(kd)=point(k)

point(k)=kd

end if

end if

end if

fi=fi + l(j)*l(j)*d(j)

l(j)=0.d0

k=j

j=point(j)

point(k)=0

end do

f(i)=fi

RETURN

END

SUBROUTINE getcgn(g)

INTEGER g

C**********************************************************************

C

C SUBROUTINE GETCGN(G)

C Get GeNerator

C

C Returns in G the number of the current random number generator

C

C

C Arguments

C

C

C G <-- Number of the current random number generator (1..32)

C INTEGER G

C

C**********************************************************************

C

INTEGER curntg,numg

SAVE curntg

PARAMETER (numg=32)

DATA curntg/1/

C

g = curntg

RETURN

ENTRY setcgn(g)

C**********************************************************************

C

C SUBROUTINE SETCGN( G )

C Set GeNerator

C

C Sets the current generator to G. All references to a generat

C are to the current generator.

C

C

C Arguments

C

C

C G --> Number of the current random number generator (1..32)

C INTEGER G

C

C**********************************************************************

C

C Abort if generator number out of range

C

IF (.NOT. (g.LT.0.OR.g.GT.numg)) GO TO 10

WRITE (*,*) ' Generator number out of range in SETCGN:',

+ ' Legal range is 1 to ',numg,' -- ABORT!'

STOP ' Generator number out of range in SETCGN'

10 curntg = g

RETURN

END

INTEGER FUNCTION ignlgi()

C**********************************************************************

C

C INTEGER FUNCTION IGNLGI()

C GeNerate LarGe Integer

C

C Returns a random integer following a uniform distribution over

C (1, 2147483562) using the current generator.

C

C This is a transcription from Pascal to Fortran of routine

C Random from the paper

C

C L'Ecuyer, P. and Cote, S. "Implementing a Random Number Package

C with Splitting Facilities." ACM Transactions on Mathematical

C Software, 17:98-111 (1991)

C

C**********************************************************************

C .. Parameters ..

INTEGER numg

PARAMETER (numg=32)

C ..

C .. Scalars in Common ..

INTEGER a1,a1vw,a1w,a2,a2vw,a2w,m1,m2

C ..

C .. Arrays in Common ..

INTEGER cg1(numg),cg2(numg),ig1(numg),ig2(numg),lg1(numg),

+ lg2(numg)

LOGICAL qanti(numg)

C ..

C .. Local Scalars ..

INTEGER curntg,k,s1,s2,z

LOGICAL qqssd

C ..

C .. External Functions ..

LOGICAL qrgnin

EXTERNAL qrgnin

C ..

C .. External Subroutines ..

EXTERNAL getcgn,inrgcm,rgnqsd,setall

C ..

C .. Common blocks ..

COMMON /globe/m1,m2,a1,a2,a1w,a2w,a1vw,a2vw,ig1,ig2,lg1,lg2,cg1,

+ cg2,qanti

C ..

C .. Save statement ..

SAVE /globe/

C ..

C .. Executable Statements ..

C

C IF THE RANDOM NUMBER PACKAGE HAS NOT BEEN INITIALIZED YET, DO SO.

C IT CAN BE INITIALIZED IN ONE OF TWO WAYS : 1) THE FIRST CALL TO

C THIS ROUTINE 2) A CALL TO SETALL.

C

IF (.NOT. (qrgnin())) CALL inrgcm()

CALL rgnqsd(qqssd)

IF (.NOT. (qqssd)) CALL setall(1234567890,123456789)

C

C Get Current Generator

C

CALL getcgn(curntg)

s1 = cg1(curntg)

s2 = cg2(curntg)

k = s1/53668

s1 = a1* (s1-k*53668) - k*12211

IF (s1.LT.0) s1 = s1 + m1

k = s2/52774

s2 = a2* (s2-k*52774) - k*3791

IF (s2.LT.0) s2 = s2 + m2

cg1(curntg) = s1

cg2(curntg) = s2

z = s1 - s2

IF (z.LT.1) z = z + m1 - 1

IF (qanti(curntg)) z = m1 - z

ignlgi = z

RETURN

END

SUBROUTINE initgn(isdtyp)

C**********************************************************************

C

C SUBROUTINE INITGN(ISDTYP)

C INIT-ialize current G-e-N-erator

C

C Reinitializes the state of the current generator

C

C This is a transcription from Pascal to Fortran of routine

C Init_Generator from the paper

C

C L'Ecuyer, P. and Cote, S. "Implementing a Random Number Package

C with Splitting Facilities." ACM Transactions on Mathematical

C Software, 17:98-111 (1991)

C

C

C Arguments

C

C

C ISDTYP -> The state to which the generator is to be set

C

C ISDTYP = -1 => sets the seeds to their initial value

C ISDTYP = 0 => sets the seeds to the first value of

C the current block

C ISDTYP = 1 => sets the seeds to the first value of

C the next block

C

C INTEGER ISDTYP

C

C**********************************************************************

C .. Parameters ..

INTEGER numg

PARAMETER (numg=32)

C ..

C .. Scalar Arguments ..

INTEGER isdtyp

C ..

C .. Scalars in Common ..

INTEGER a1,a1vw,a1w,a2,a2vw,a2w,m1,m2

C ..

C .. Arrays in Common ..

INTEGER cg1(numg),cg2(numg),ig1(numg),ig2(numg),lg1(numg),

+ lg2(numg)

LOGICAL qanti(numg)

C ..

C .. Local Scalars ..

INTEGER g

C ..

C .. External Functions ..

LOGICAL qrgnin

INTEGER mltmod

EXTERNAL qrgnin,mltmod

C ..

C .. External Subroutines ..

EXTERNAL getcgn

C ..

C .. Common blocks ..

COMMON /globe/m1,m2,a1,a2,a1w,a2w,a1vw,a2vw,ig1,ig2,lg1,lg2,cg1,

+ cg2,qanti

C ..

C .. Save statement ..

SAVE /globe/

C ..

C .. Executable Statements ..

C Abort unless random number generator initialized

IF (qrgnin()) GO TO 10

WRITE (*,*) ' INITGN called before random number generator ',

+ ' initialized -- abort!'

STOP ' INITGN called before random number generator initialized'

10 CALL getcgn(g)

IF ((-1).NE. (isdtyp)) GO TO 20

lg1(g) = ig1(g)

lg2(g) = ig2(g)

GO TO 50

20 IF ((0).NE. (isdtyp)) GO TO 30

CONTINUE

GO TO 50

C do nothing

30 IF ((1).NE. (isdtyp)) GO TO 40

lg1(g) = mltmod(a1w,lg1(g),m1)

lg2(g) = mltmod(a2w,lg2(g),m2)

GO TO 50

40 STOP 'ISDTYP NOT IN RANGE'

50 cg1(g) = lg1(g)

cg2(g) = lg2(g)

RETURN

END

SUBROUTINE inrgcm()

C**********************************************************************

C

C SUBROUTINE INRGCM()

C INitialize Random number Generator CoMmon

C

C

C Function

C

C

C Initializes common area for random number generator. This saves

C the nuisance of a BLOCK DATA routine and the difficulty of

C assuring that the routine is loaded with the other routines.

C

C**********************************************************************

C .. Parameters ..

INTEGER numg

PARAMETER (numg=32)

C ..

C .. Scalars in Common ..

INTEGER a1,a1vw,a1w,a2,a2vw,a2w,m1,m2

C ..

C .. Arrays in Common ..

INTEGER cg1(numg),cg2(numg),ig1(numg),ig2(numg),lg1(numg),

+ lg2(numg)

LOGICAL qanti(numg)

C ..

C .. Local Scalars ..

INTEGER i

LOGICAL qdum

C ..

C .. External Functions ..

LOGICAL qrgnsn

EXTERNAL qrgnsn

C ..

C .. Common blocks ..

COMMON /globe/m1,m2,a1,a2,a1w,a2w,a1vw,a2vw,ig1,ig2,lg1,lg2,cg1,

+ cg2,qanti

C ..

C .. Save statement ..

SAVE /globe/

C ..

C .. Executable Statements ..

C V=20; W=30;

C

C A1W = MOD(A1**(2**W),M1) A2W = MOD(A2**(2**W),M2)

C A1VW = MOD(A1**(2**(V+W)),M1) A2VW = MOD(A2**(2**(V+W)),M2)

C

C If V or W is changed A1W, A2W, A1VW, and A2VW need to be recomputed.

C An efficient way to precompute a**(2*j) MOD m is to start with

C a and square it j times modulo m using the function MLTMOD.

C

m1 = 2147483563

m2 = 2147483399

a1 = 40014

a2 = 40692

a1w = 1033780774

a2w = 1494757890

a1vw = 2082007225

a2vw = 784306273

DO 10,i = 1,numg

qanti(i) = .FALSE.

10 CONTINUE

C

C Tell the world that common has been initialized

C

qdum = qrgnsn(.TRUE.)

RETURN

END

INTEGER FUNCTION mltmod(a,s,m)

C**********************************************************************

C

C INTEGER FUNCTION MLTMOD(A,S,M)

C

C Returns (A*S) MOD M

C

C This is a transcription from Pascal to Fortran of routine

C MULtMod_Decompos from the paper

C

C L'Ecuyer, P. and Cote, S. "Implementing a Random Number Package

C with Splitting Facilities." ACM Transactions on Mathematical

C Software, 17:98-111 (1991)

C

C

C Arguments

C

C

C A, S, M -->

C INTEGER A,S,M

C

C**********************************************************************

C .. Parameters ..

INTEGER h

PARAMETER (h=32768)

C ..

C .. Scalar Arguments ..

INTEGER a,m,s

C ..

C .. Local Scalars ..

INTEGER a0,a1,k,p,q,qh,rh

C ..

C .. Executable Statements ..

C

C H = 2**((b-2)/2) where b = 32 because we are using a 32 bit

C machine. On a different machine recompute H

C

IF (.NOT. (a.LE.0.OR.a.GE.m.OR.s.LE.0.OR.s.GE.m)) GO TO 10

WRITE (*,*) ' A, M, S out of order in MLTMOD - ABORT!'

WRITE (*,*) ' A = ',a,' S = ',s,' M = ',m

WRITE (*,*) ' MLTMOD requires: 0 < A < M; 0 < S < M'

STOP ' A, M, S out of order in MLTMOD - ABORT!'

10 IF (.NOT. (a.LT.h)) GO TO 20

a0 = a

p = 0

GO TO 120

20 a1 = a/h

a0 = a - h*a1

qh = m/h

rh = m - h*qh

IF (.NOT. (a1.GE.h)) GO TO 50

a1 = a1 - h

k = s/qh

p = h* (s-k*qh) - k*rh

30 IF (.NOT. (p.LT.0)) GO TO 40

p = p + m

GO TO 30

40 GO TO 60

50 p = 0

C

C P = (A2*S*H)MOD M

C

60 IF (.NOT. (a1.NE.0)) GO TO 90

q = m/a1

k = s/q

p = p - k* (m-a1*q)

IF (p.GT.0) p = p - m

p = p + a1* (s-k*q)

70 IF (.NOT. (p.LT.0)) GO TO 80

p = p + m

GO TO 70

80 CONTINUE

90 k = p/qh

C

C P = ((A2*H + A1)*S)MOD M

C

p = h* (p-k*qh) - k*rh

100 IF (.NOT. (p.LT.0)) GO TO 110

p = p + m

GO TO 100

110 CONTINUE

120 IF (.NOT. (a0.NE.0)) GO TO 150

C

C P = ((A2*H + A1)*H*S)MOD M

C

q = m/a0

k = s/q

p = p - k* (m-a0*q)

IF (p.GT.0) p = p - m

p = p + a0* (s-k*q)

130 IF (.NOT. (p.LT.0)) GO TO 140

p = p + m

GO TO 130

140 CONTINUE

150 mltmod = p

C

RETURN

END

LOGICAL FUNCTION qrgnin()

C**********************************************************************

C

C LOGICAL FUNCTION QRGNIN()

C Q Random GeNerators INitialized?

C

C A trivial routine to determine whether or not the random

C number generator has been initialized. Returns .TRUE. if

C it has, else .FALSE.

C

C**********************************************************************

C .. Scalar Arguments ..

LOGICAL qvalue

C ..

C .. Local Scalars ..

LOGICAL qinit

C ..

C .. Entry Points ..

LOGICAL qrgnsn

C ..

C .. Save statement ..

SAVE qinit

C ..

C .. Data statements ..

DATA qinit/.FALSE./

C ..

C .. Executable Statements ..

qrgnin = qinit

RETURN

ENTRY qrgnsn(qvalue)

C**********************************************************************

C

C LOGICAL FUNCTION QRGNSN( QVALUE )

C Q Random GeNerators Set whether iNitialized

C

C Sets state of whether random number generator is initialized

C to QVALUE.

C

C This routine is actually an entry in QRGNIN, hence it is a

C logical function. It returns the (meaningless) value .TRUE.

C

C**********************************************************************

qinit = qvalue

qrgnsn = .TRUE.

RETURN

END

REAL FUNCTION ranf()

C**********************************************************************

C

C REAL FUNCTION RANF()

C RANDom number generator as a Function

C

C Returns a random floating point number from a uniform distribution

C over 0 - 1 (endpoints of this interval are not returned) using the

C current generator

C

C This is a transcription from Pascal to Fortran of routine

C Uniform_01 from the paper

C

C L'Ecuyer, P. and Cote, S. "Implementing a Random Number Package

C with Splitting Facilities." ACM Transactions on Mathematical

C Software, 17:98-111 (1991)

C

C**********************************************************************

C .. External Functions ..

INTEGER ignlgi

EXTERNAL ignlgi

C ..

C .. Executable Statements ..

C

C 4.656613057E-10 is 1/M1 M1 is set in a data statement in IGNLGI

C and is currently 2147483563. If M1 changes, change this also.

C

ranf = ignlgi()*4.656613057E-10

RETURN

END

SUBROUTINE setall(iseed1,iseed2)

C**********************************************************************

C

C SUBROUTINE SETALL(ISEED1,ISEED2)

C SET ALL random number generators

C

C Sets the initial seed of generator 1 to ISEED1 and ISEED2. The

C initial seeds of the other generators are set accordingly, and

C all generators states are set to these seeds.

C

C This is a transcription from Pascal to Fortran of routine

C Set_Initial_Seed from the paper

C

C L'Ecuyer, P. and Cote, S. "Implementing a Random Number Package

C with Splitting Facilities." ACM Transactions on Mathematical

C Software, 17:98-111 (1991)

C

C

C Arguments

C

C

C ISEED1 -> First of two integer seeds

C INTEGER ISEED1

C

C ISEED2 -> Second of two integer seeds

C INTEGER ISEED1

C

C**********************************************************************

C .. Parameters ..

INTEGER numg

PARAMETER (numg=32)

C ..

C .. Scalar Arguments ..

INTEGER iseed1,iseed2

LOGICAL qssd

C ..

C .. Scalars in Common ..

INTEGER a1,a1vw,a1w,a2,a2vw,a2w,m1,m2

C ..

C .. Arrays in Common ..

INTEGER cg1(numg),cg2(numg),ig1(numg),ig2(numg),lg1(numg),

+ lg2(numg)

LOGICAL qanti(numg)

C ..

C .. Local Scalars ..

INTEGER g,ocgn

LOGICAL qqssd

C ..

C .. External Functions ..

INTEGER mltmod

LOGICAL qrgnin

EXTERNAL mltmod,qrgnin

C ..

C .. External Subroutines ..

EXTERNAL getcgn,initgn,inrgcm,setcgn

C ..

C .. Common blocks ..

COMMON /globe/m1,m2,a1,a2,a1w,a2w,a1vw,a2vw,ig1,ig2,lg1,lg2,cg1,

+ cg2,qanti

C ..

C .. Save statement ..

SAVE /globe/,qqssd

C ..

C .. Data statements ..

DATA qqssd/.FALSE./

C ..

C .. Executable Statements ..

C

C TELL IGNLGI, THE ACTUAL NUMBER GENERATOR, THAT THIS ROUTINE

C HAS BEEN CALLED.

C

qqssd = .TRUE.

CALL getcgn(ocgn)

C

C Initialize Common Block if Necessary

C

IF (.NOT. (qrgnin())) CALL inrgcm()

ig1(1) = iseed1

ig2(1) = iseed2

CALL initgn(-1)

DO 10,g = 2,numg

ig1(g) = mltmod(a1vw,ig1(g-1),m1)

ig2(g) = mltmod(a2vw,ig2(g-1),m2)

CALL setcgn(g)

CALL initgn(-1)

10 CONTINUE

CALL setcgn(ocgn)

RETURN

ENTRY rgnqsd(qssd)

C**********************************************************************

C

C SUBROUTINE RGNQSD

C Random Number Generator Query SeeD set?

C

C Returns (LOGICAL) QSSD as .TRUE. if SETALL has been invoked,

C otherwise returns .FALSE.

C

C**********************************************************************

qssd = qqssd

RETURN

END
